# Lentiviral mediated *RPE65* gene transfer in healthy hiPSCs-derived retinal pigment epithelial cells markedly increased *RPE65 mRNA*, but modestly protein level

**DOI:** 10.1038/s41598-020-65657-y

**Published:** 2020-06-01

**Authors:** Florian Udry, Sarah Decembrini, David M. Gamm, Nicole Déglon, Corinne Kostic, Yvan Arsenijevic

**Affiliations:** 1 Department of ophthalmology, Unit of Retinal Degeneration and Regeneration, University of Lausanne, Hôpital ophtalmique Jules-Gonin, 1004 Lausanne, Switzerland; 2 McPherson Eye Research Institute, Waisman Center and Department of Ophthalmology and Visual Sciences, and University of Wisconsin-Madison, Madison, USA; 3 Neuroscience Research Center, Laboratory of Neurotherapies and Neuromodulation, Lausanne University Hospital and University of Lausanne, Lausanne, Switzerland; 4 Department of Biomedicine, University Hospital Basel & University Basel, Hebelstr. 20, 4031 Basel, Switzerland

**Keywords:** Visual system, Stem-cell differentiation

## Abstract

The retinal pigment epithelium (RPE) is a monolayer of cobblestone-like epithelial cells that accomplishes critical functions for the retina. Several protocols have been published to differentiate pluripotent stem cells into RPE cells suitable for disease modelling and therapy development. In our study, the RPE identity of human induced pluripotent stem cell (hiPSC)-derived RPE (iRPE) was extensively characterized, and then used to test a lentiviral-mediated *RPE65* gene augmentation therapy. A dose study of the lentiviral vector revealed a dose-dependent effect of the vector on *RPE65* mRNA levels. A marked increase of the *RPE65* mRNA was also observed in the iRPE (100-fold) as well as in an experimental set with RPE derived from another hiPSC source and from foetal human RPE. Although iRPE displayed features close to bona fide RPE, no or a modest increase of the RPE65 protein level was observed depending on the protein detection method. Similar results were observed with the two other cell lines. The mechanism of RPE65 protein regulation remains to be elucidated, but the current work suggests that high vector expression will not produce an excess of the normal RPE65 protein level.

## Introduction

Lying between the outer retina and the choroidal vessels, the retinal pigment epithelium (RPE) fulfils many crucial tasks for visual function such as phagocytosis, 11-*cis* retinal recycling, ion and water transports, and growth factor secretion^1^. Deficiencies in RPE functions give rise to various diseases such as Leber congenital amaurosis^2^ (LCA) or retinitis pigmentosa^3^, most of which result in drastic visual impairments or blindness. The generation of RPE *in vitro* is particularly valuable to understand pathophysiological mechanisms through disease modelling and to help answer the major challenge of developing therapies.

Numerous protocols to differentiate embryonic or induced-pluripotent stem cells (ESCs or iPSCs, respectively) have been published^4–6^, providing researchers with a renewable source of RPE. Thanks to the protocols developed, the therapeutic potential of stem cell-derived RPE cells has been explored by transplantation in RPE degenerative diseases, first in animal models^5,7,8^, and more recently in human patients suffering from age-related macular degeneration^9–12^ (AMD) or Stargardt’s disease^13^.

In addition, iPSC-derived RPE (iRPE) has been used as an *in vitro* model for Best disease^14^ and choroideremia^15^. It has proven to be an interesting alternative model for some diseases such as AMD^16^, where no animal recapitulates all the cellular features of human AMD^17^.

Worthy of note, more and more genetic diseases are tackled with a gene therapy approach and iRPE has also allowed to test the efficacy of adeno-associated virus (AAV)- or CRISPR-mediated gene correction^15,16,18^.

Moreover, in many diseases that are candidates for gene augmentation therapy, it is not clear how many vector copies are necessary to re-establish a physiological level of gene expression. For instance, AAV-mediated gene augmentation therapy on *RPE65*-deficient dogs showed a significant improvement in visual function that appeared on electroretinogram readings, but this success could not be reproduced on LCA2 patients with similar doses^19–22^. Therefore, studying the vector dose-response relationship in human iRPE may help to better estimate the vector dose required to attain optimal rescue in human *in vivo*.

In our study, we evaluated the suitability of iRPE cells as an *in vitro* model for lentiviral gene therapy, first through an extensive characterization of RPE features, followed by testing different lentiviral constructs on iRPE cells. We subjected healthy iRPE to an *RPE65* gene augmentation therapy previously tested on *RPE65* deficiency mouse models^23,24^ and healthy non-human primates^25^ to study *RPE65* gene and protein expression.

## Results

### From hiPSCs to RPE

#### Differentiation protocol

**Figure 1:**
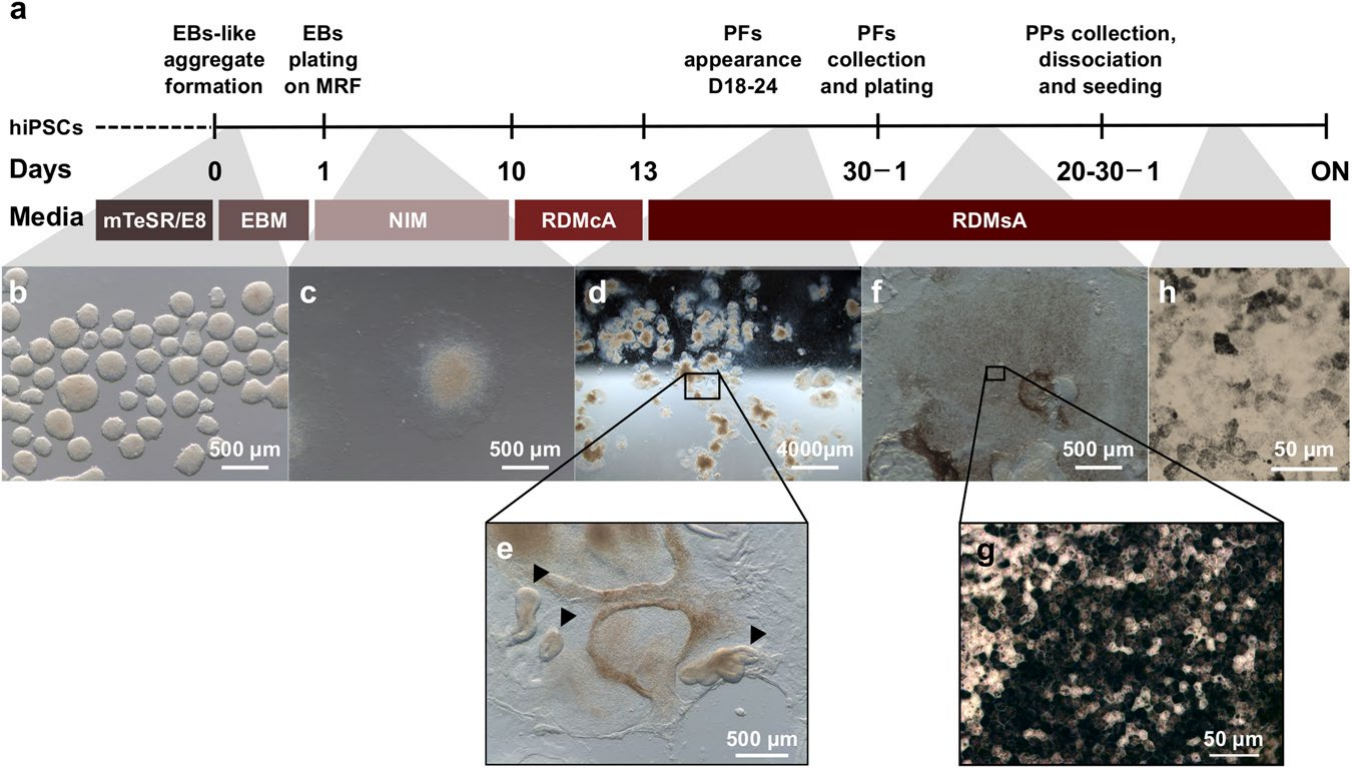
RPE differentiation protocol timeline. (**a**) Schematic depiction of the differentiation protocol highlighting the time course and media used. The protocol starts with hiPSCs in feeder-free condition that are lifted in clumps using collagenase to form (**b**) embryoid body-like aggregates (EB) in floating condition in an embryoid body media (EBM). EBs are plated on day 1 on Matrigel Reduced Factor (MRF) in a neural induction media (NIM) and (**c**) expand. The cells are switched to a retinal differentiation medium with vitamin A (RDMcA) from day 10 to 13, then without vitamin A (RDMsA). (**d**,**e**) Pigmented foci (PFs) appear around day 18 to 24 and are collected on day 30. Optic vesicle-like structures can also be observed (e, arrowheads). (**f**,**g**) Plated PFs develop pigmented patches (PPs) that are collected, dissociated and seeded to obtain (**h**) the pure hiPSC-derived RPE (iRPE) cell monolayer.

We first tested 3 hiPSC lines for their potency to generate iRPE: two of them produced 10 to 50 pigmented foci during the differentiation protocol, whereas the one described by Singh and colleagues^14^ produced 50 to 200 foci. We thus decided to focus the iRPE characterization on this hiPSC line. The differentiation protocol was adapted from Singh *et al*.^14^, recapitulating its main steps with modifications in time points (plating at day 1) and minor changes in the media (addition of embryoid body media – EBM and retinal differentiation media cum vitamin A - RDMcA) (Fig. 1a). Human iPSCs were lifted and placed in free-floating condition to form embryoid body-like aggregates (Fig. 1b) that were plated one day later to expand (Fig. 1c) in Neural Induction Medium (NIM) which contains a high concentration of insulin potentially mimicking IGF1 action^26,27^. The medium was switched 9 days later to a Retinal Differentiation Medium (RDM) in order to commit cells to the retinal fate possibly by the stimulation of the retinoic acid^28^ and thyroid hormone^29^.

In our hands, a short induction in RDMcA resulted in quicker pigmentation that may originate from the retinoic acid (RA). The RA produced from the vitamin A in the RDMcA may help to create the optic vesicle presumptive fields that later form the retina and RPE. However, after 3 days, the medium is changed to a vitamin A-free RDM (RDMsA) to prevent the neural retina fate^30^ that may arise detrimentally to RPE differentiation due to the trans-differentiation ability of RPE during development^31^.

Pigmented foci (Fig. 1d,e) appeared around day 18-24 and were manually collected around day 30 to be re-plated as a purification and enrichment step. The pigmented foci developed large pigmented patches reaching up to several millimetres of diameter (Fig. 1e), composed of iRPE cells harbouring the typical RPE cobblestone morphology (Fig. 1f). After 20 to 30 days of expansion, the pigmented patches were dissected, dissociated and plated. This step is considered as passage 1 (P1). All assays performed were on P3 iRPE matured 6 weeks on transwell (Fig. 1h) to allow time for the cells to re-acquire RPE characteristics lost during the passages (as previously observed in ESC-derived RPE^32,33^).

#### Expression of RPE gene markers

**Figure 2:**
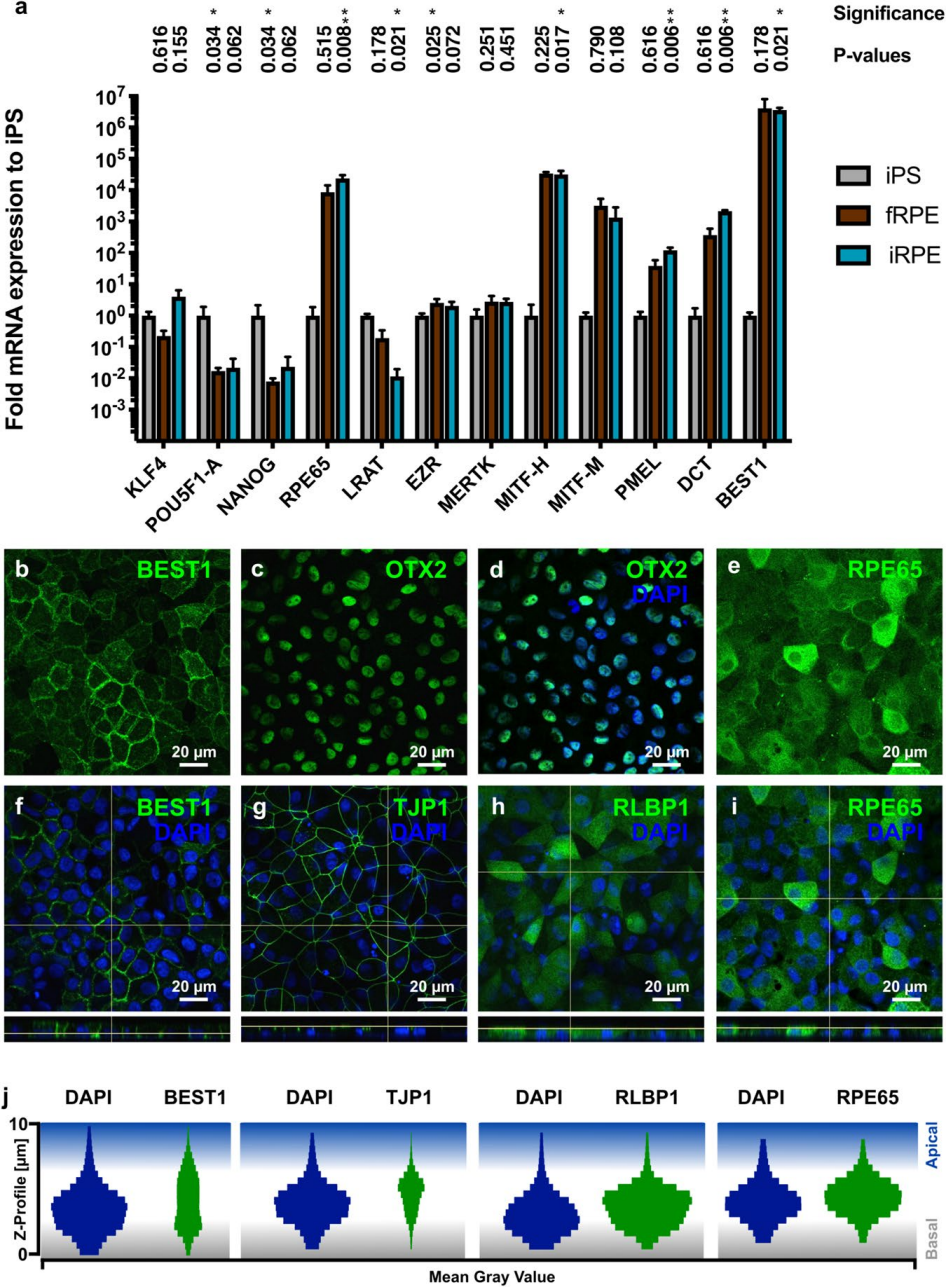
Expression of RPE markers at mRNA and protein levels. (**a**) Quantitative PCR investigating RPE marker expression in fRPE (n = 3) and iRPE (n = 6) normalized to iPS (n = 3). Bars represent mean ± SD. Kruskal-Wallis tests were performed (iPS vs. iRPE and iPS vs. fRPE for each gene) followed by Dunn’s multiple comparisons test. Significance threshold: *<0.05; **<0.01. (**b**,**e**) Z-stack Maximum Intensity Projection of iRPE cells labelled with anti-BEST1 and -RPE65 antibodies, respectively. (**c**,**d**) iRPE cells immunolabelled for OTX2. (**f**–**i**) Z-stacks acquired over the whole iRPE cell monolayers to investigate RPE marker localizations. Orthogonal view confirmed the preferential basolateral or apical distributions of BEST1 (**f**) and ZO-1 proteins (**g**), respectively. RPE65 and CRALBP localizations were widespread in iRPE cells (**h**,**i**). (**j**) Violin plots corresponding to images (**f**–**i**) showing the distribution of the labelled proteins and DAPI staining throughout the Z-stacks. Images are representative of 1-2 observations per marker in at least 3 iRPE lines.

In order to confirm the RPE identity of the iRPE, we investigated the RPE gene markers’ expression compared to iPSCs and cultured foetal RPE (fRPE). The RT-qPCR results (Fig. 2a) showed a marked and significant upregulation of *RPE65*, *MITF-H* (RPE expressed isoform H), *PMEL*, *DCT* and *BEST1* gene expression in iRPE compared to iPSCs while there was no significant difference for *KLF4*, *EZR*, *MERTK* and *MITF-M* (melanocyte specific isoform M). A significant downregulation for the pluripotency markers *POU5F1-A* and *NANOG* was observed. Nonetheless, the iRPE was still positive for these markers (<30 Cq).

Surprisingly, a downregulation of *LRAT* expression in iRPE compared to iPSCs was observed whereas an increase similar to *RPE65* was expected, as both are involved in the visual cycle^34^. Nonetheless *LRAT* mRNA expression can be considered positive in iRPE (<30 Cq).

Despite this unexpected result, the iRPE showed an expression pattern close to fRPE with a strong significant correlation (R = 0.902) for the 10 genes investigated (Supp. Fig. 1b). Note that *LRAT*, *EZR* and *MERTK* in fRPE have similar expression levels in comparison to iRPE.

At the protein level, the RPE-specific proteins RPE65, RLBP1 (also known as CRALBP), BEST1, TJP1 (also known as ZO-1) and OTX2 were all expressed although with some heterogeneity (Fig. 1b–i). NANOG was not expressed. However, a few POU5F1-A positive cells were observed (Supp. Fig. 2). Quantification of OTX2 positive cells (Supp. Fig. 2) showed that more than 94% of cells express OTX2 confirming a homogeneous RPE identity. Moreover, the polarization of some critical proteins was confirmed by confocal microscopy, particularly the apical distribution of ZO-1 and the basolateral presence of BEST1 as assessed by orthogonal view (Fig. 1f–i). In order to better visualize the protein distribution in the cell in the Z-axis, the mean grey value of each Z-stack of Fig. 1f–i was measured to present the Z-profile of both the nucleus (labelled by DAPI) and the protein of interest (Fig. 1j).

As a comparison, fRPE showed similar staining and protein localization (Supp. Fig. 3). However, after cell passages, the cells lose progressively their identity (Supp. Fig. 7). The iRPE coming from replicated differentiation experiment displayed consistent results (Supp. Fig. 4).

#### Cell ultrastructure analysis reveals polarized distributions of certain organelles

**Figure 3:**
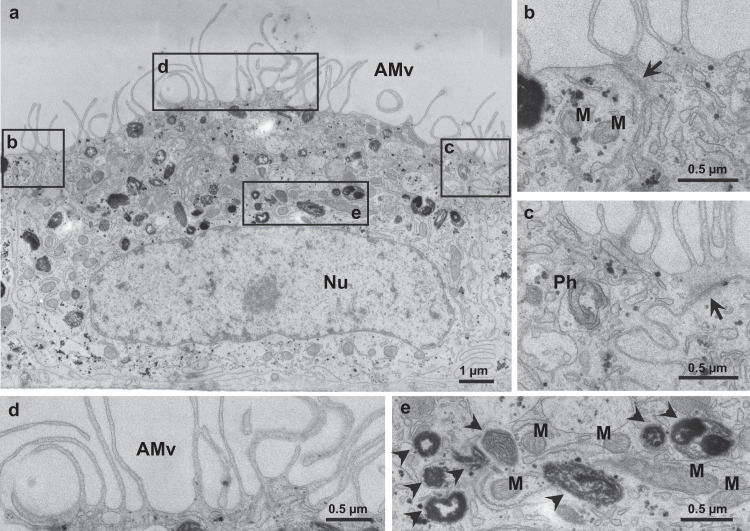
iRPE cells present characteristic ultrastructure features of human adult RPE (**a**) Typical iRPE cell ultrastructure with a large nucleus in the basal part of the cell and with dense structures in the apical region containing microvilli. (**c**–**e**) Inserts show these iRPE cell features with high magnification. (**b**,**c**) Tight junctions (arrows) between two iRPE cells. (**d**) Multiple apical microvilli (AMv) are present at the cell surface. (**e**) Melanosomes (arrowheads) at different maturation stages were observed. Nu, Nucleus; Ph, Phagosome; M, Mitochondria.

Vertical sections of iRPE cells were imaged by electron microscopy to reveal their ultrastructure (Fig. 3a). The presence of tight junctions between iRPE cells (Fig. 3b,c), apical microvilli (Fig. 3d), as well as a basal nucleus and melanosomes at different stages of maturation^35^ (Fig. 3e) confirmed their RPE identity.

#### Differentiated iRPE form an epithelium with high cohesion

**Figure 4:**
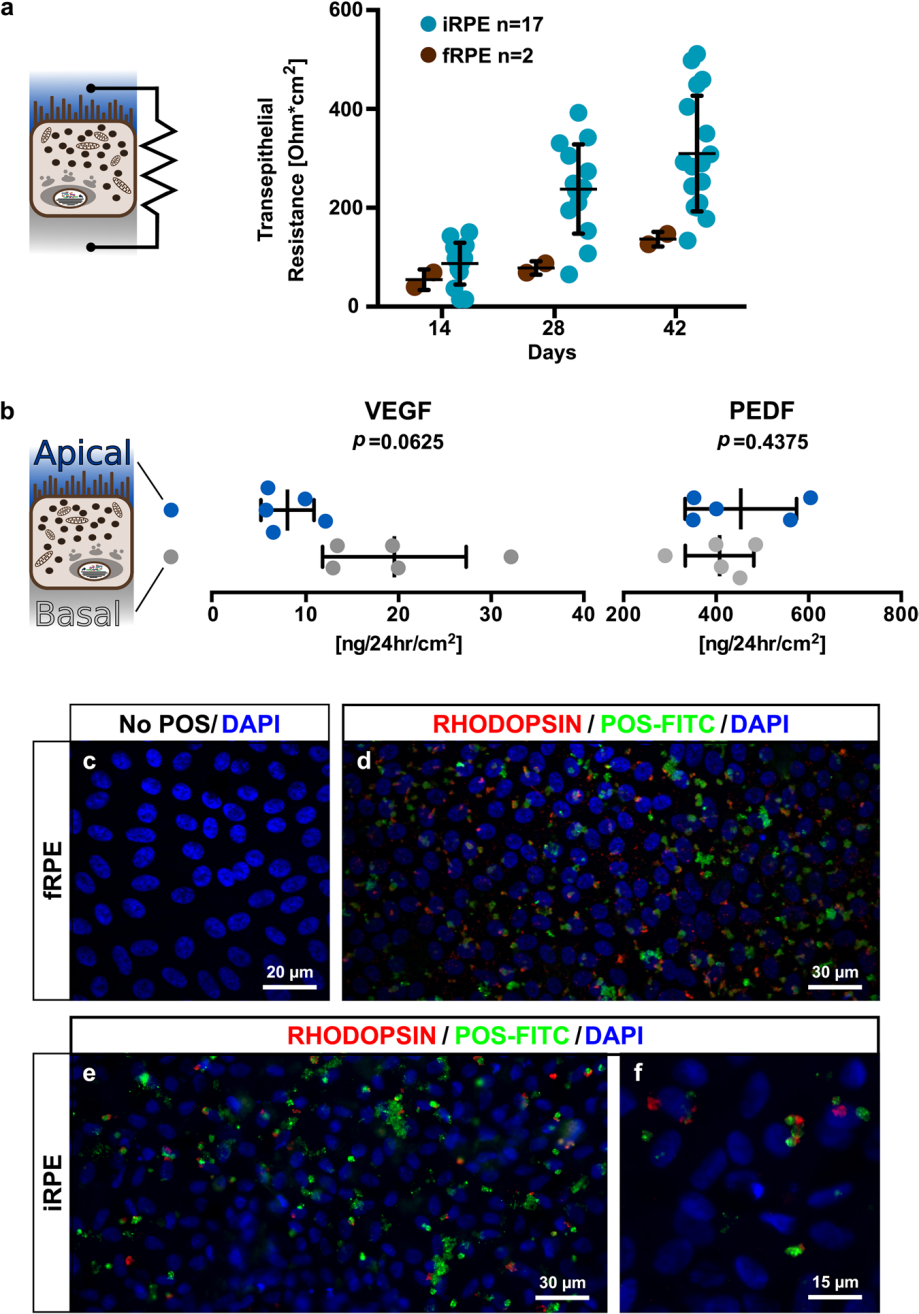
After maturation, iRPE cells present typical biological functions of RPE cells. (**a**) TER was measured on iRPE and fRPE monolayers at 14, 28 and 42 days in culture on transwell. Bars represents mean ± SD. (**b**) PEDF and VEGF secretion levels were measured by ELISA after 42 days in culture for both apical (blue) and basal (grey) compartments of n = 5 iRPE lines. Comparison between compartments was done by paired Wilcoxon match-pairs signed-rank test. Bars represent mean ± SD. (**c**–**f**) FITC-conjugated POS were fed to iRPE and fRPE cells for 16 h and the RHODOPSIN protein was immunolabelled without permeabilization (red labelling). As a result, the internalized POS fluoresce in green only, and microvilli-bound POS fluoresce in green and red. *p*: p-value. P-value significance threshold for all tests: 0.05.

As part of the blood retinal barrier^1^, RPE cells need to form a functional epithelium that can regulate ion, molecule and protein transport. By measuring the transepithelial resistance, we sought to investigate the epithelium integrity. The iRPE monolayer showed an increasing transepithelial resistance in parallel with the number of days in culture (Fig. 4a) with a mean transepithelial resistance of around 300 Ohm*cm^2^ at day 42 (when characterization is performed), which is in line with previous publications on iRPE^14,18,36–38^ and above the functional minimum required as stated by others^39,40^. Foetal RPE displayed a limited transepithelial resistance reaching approximately 150 Ohm*cm^2^ at day 42, confirming results obtained by Takahashi’s lab with fRPE from the same source^38^. However, fRPE from a different source reached a higher transepithelial resistance^14,39^, suggesting heterogeneity in fRPE lines that may stem from the age of the donor, isolation process or culture conditions.

#### VEGF is preferentially secreted at the basal iRPE side

Factor secretion is another important feature of RPE cells^1^. Many proteins have been shown to be secreted by the RPE such as the vascular endothelial or pigment epithelium growth factor (VEGF and PEDF, respectively). To determine the secretive ability of the iRPE, the levels of both VEGF and PEDF were measured by ELISA (Fig. 4b) in the medium of the apical and basal compartments of transwells 24 hr after changing the medium. VEGF was preferentially secreted towards the basal compartments of the transwell, which corresponds to the choroid *in vivo*, as expected from previous publications on stem cell-derived RPE^14,38,41–43^ or adult human RPE^44^. On the other hand, although PEDF secretion level was comparable to previous reports^14,36,38,43^, it was not observed in a polarized fashion.

#### iRPE cells phagocytize bovine photoreceptor outer segments

*In vivo*, the RPE phagocytizes debris coming from the daily partial renewal of photoreceptor outer segments (POS)^45^. The internalization mechanism of the POS has been identified and it has been shown that the integrins involved in POS internalization are highly conserved between bovine and human^46^, allowing to test this particular pathway and not only observe passive uptake of the POS. To assess the iRPE phagocytic capacity, we fed fRPE and iRPE with 5 million bovine POS over 16 hr and performed the same treatment with fRPE in parallel as a comparison point. Both fRPE and iRPE were able to phagocytize bovine POS (Fig. 4c–f), although POS uptake was heterogeneous.

#### Lentiviral-mediated transduction of iRPE cells

**Figure 5:**
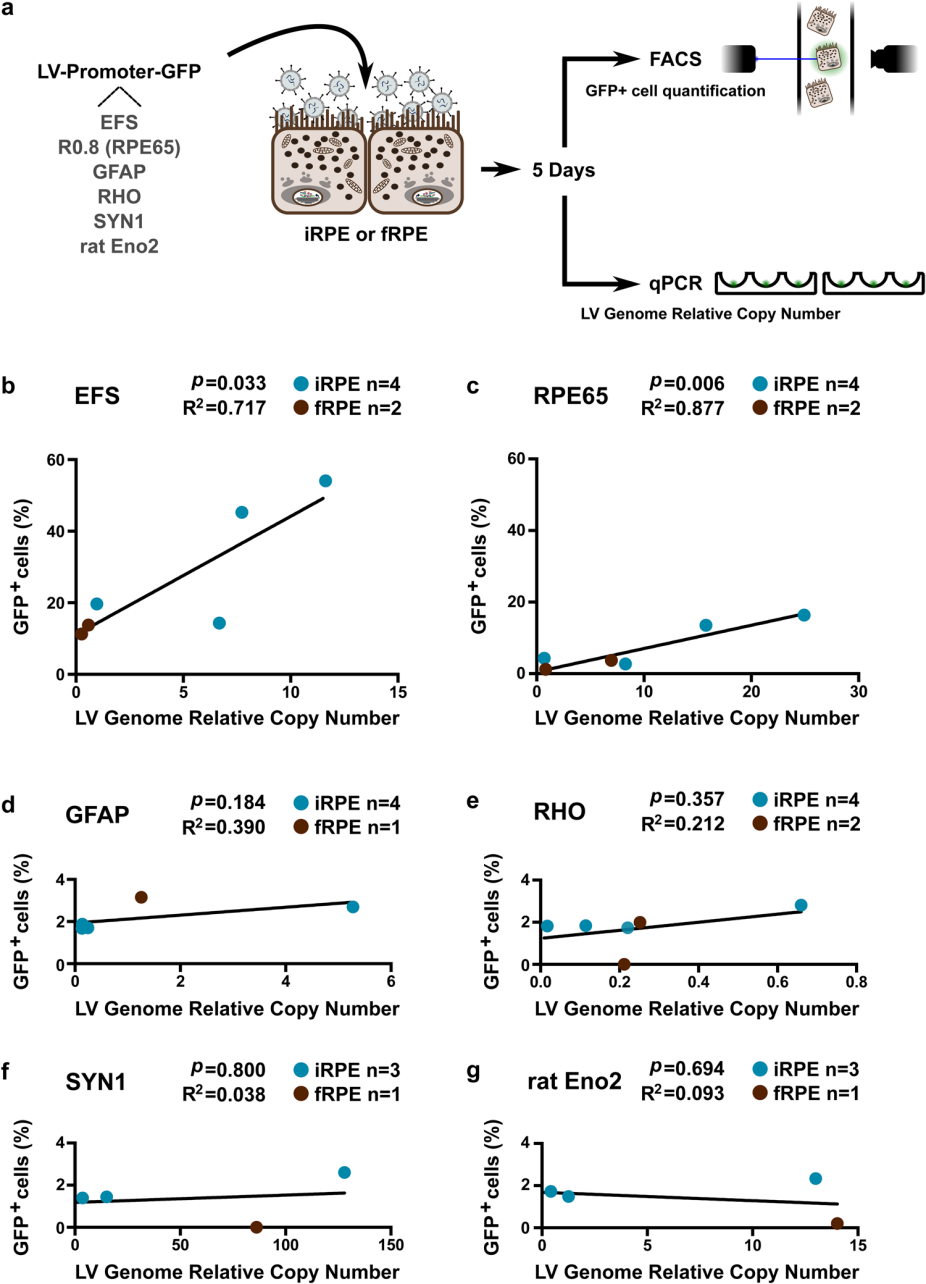
GFP reporter gene expressions by lentiviral vector transduction reveals iRPE cells specificity. (**a**) Six different lentiviral vector constructs were produced bearing a GFP under ubiquitous (EFS), RPE specific (R0.8 = partial *RPE65* promoter) or retinal but RPE aspecific (RHODOPSIN), glial (GFAP) or neural (SYNAPSIN and rat NSE) promoter. iRPE and fRPE cells were transduced using these vectors and the proportion of GFP-positive iRPE and fRPE cells was determined by FACS. In addition, the relative lentiviral genome copy number was quantified by qPCR. (**b**,**c**) EFS and R0.8 promoters showed a significant positive correlation between the percentage of GFP-positive cells (iRPE and fRPE together) and the relative lentiviral genome copy number whereas none of the aspecific promoters for RPE (**d**–**g**) did. R^2^: coefficient of determination of the linear regression. *p*: p-value from the F-test under the assumption of a null slope. P-value significance threshold: 0.05.

Lentiviral vectors (LV) are suitable vectors to efficiently deliver a transgene in the RPE^47,48^ and to carry long construct containing either long transgene or complex regulatory sequences^49,50^. We thus tested LV in the human iRPE to evaluate the bona fide RPE identity of the iRPE as well as confirm the tropism of the lentiviral vector for the human RPE as observed *in vivo* in rodent^24,47^. We thus transduced iRPE and fRPE with 6 different lentiviral vectors (Fig. 5a) each bearing a construct to express GFP driven by different promoters: either RPE-specific (*RPE65* short sequence: R0.8)^20^, ubiquitous (*EFS*) or specific for glial, photoreceptor or neuronal cells (*GFAP*, *RHO*, *SYN1* and rat *Eno2* respectively). To ensure that the lentiviral vectors transduced the cells, we determined by qPCR the lentiviral genome copy number relative to the iRPE or fRPE *ACTB* gene copies. All the vectors successfully transduced the cell, as revealed by the positive qPCR for the lentiviral genome amplification. To assess the activity of the transduced promoters, the percentage of GFP^+^ cells was linearly regressed to the lentiviral genome relative copy number. The ubiquitous *EFS* and specific R0.8 promoters showed a substantial prediction of the percentage of GFP^+^ cells by the lentiviral genome relative copy number (Fig. 5b,c) whereas none of the nonspecific promoters for RPE did, as assessed by the R^2^ coefficient of determination. In addition, only the *EFS* and R0.8 promoters presented significantly positive slopes of the best-fit line equations.

#### *RPE65* gene augmentation by lentiviral gene therapy

**Figure 6:**
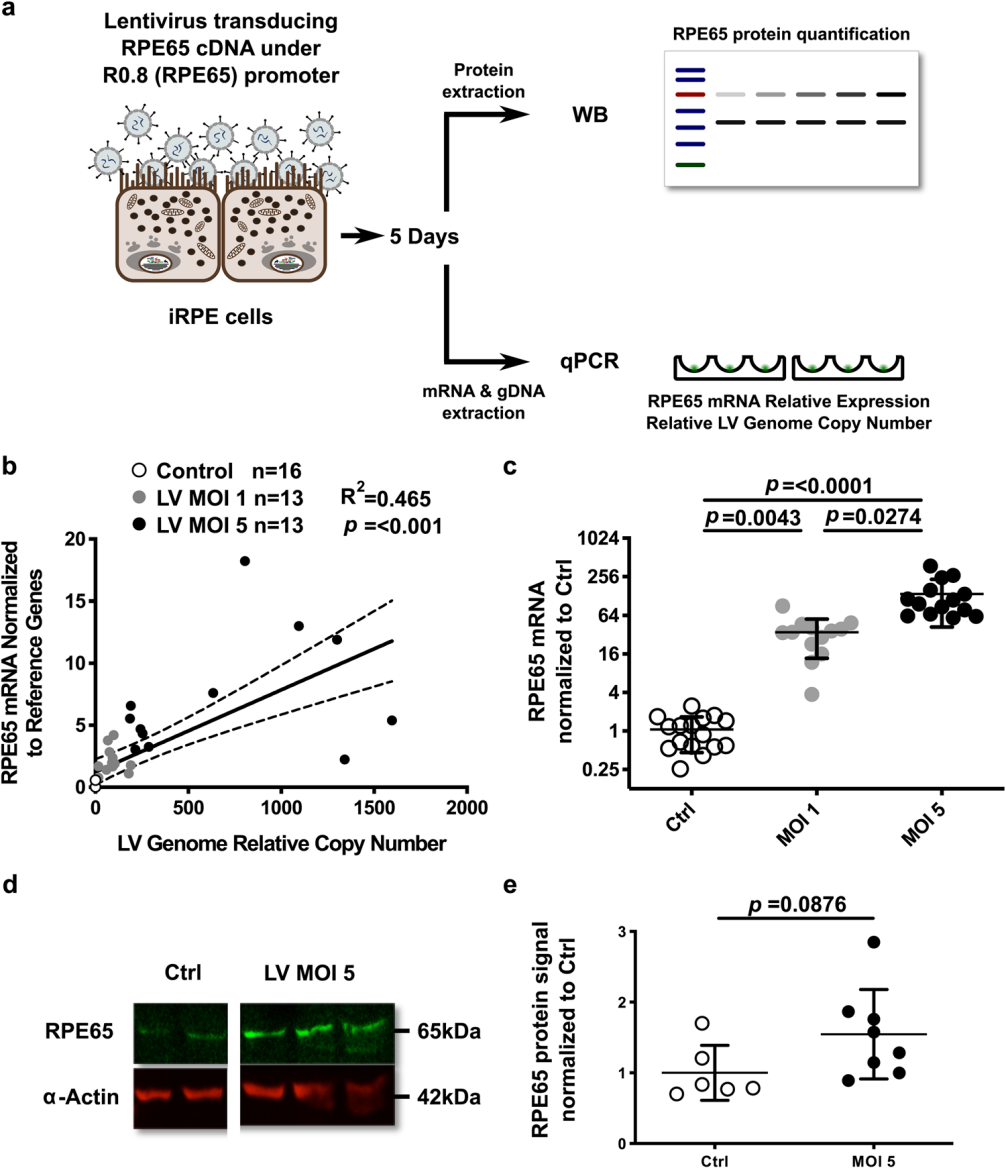
Lentivirus-mediated *RPE65* gene augmentation expression. (**a**) Schematic of iRPE cells transduced with a lentivirus expressing *RPE65* cDNA under the control of the R0.8 partial *RPE65* promoter. Lentiviral genome relative copy number, relative *RPE65* mRNA and protein expressions were quantified five days later. (**b**) The linear regression between the relative lentiviral genome copy number and *RPE65* mRNA relative expression showed a positive correlation. Dashed lines represent the 95% confidence interval of the regression curve. R^2^: coefficient of determination of the linear regression. *p*: p-value from the F-test under the assumption of a null slope. P-value significance threshold: 0.05. (**c**) Comparison of normalized *RPE65* mRNA expressions between untreated control (Ctrl) and iRPE cells transduced with R0.8-RPE65 lentivirus at multiplicity of infection (MOI) 1 or 5. *p*: p-value of Dunn’s multiple comparisons test after Kruskal-Wallis test. P-value significance threshold: 0.05. Data are expressed as mean ± SD. (**d**) Representative near infra-red imaging of RPE65 and α-Actin proteins on immunoblot. (**e**) Western blot densitometry analysis of RPE65 protein level normalized to α-Actin between untreated (Ctrl) and R0.8-RPE65 lentivirus-transduced iRPE cells (MOI 5). Mean ± SD. Unpaired t-test p-value significance threshold: 0.05.

Finally, we investigated the response of the iRPE to a *RPE65* gene augmentation treatment that has shown rod and cone function restoration in mouse models of *Rpe65* deficiency^23,24^ and is also manageable to use in non-human primate retina^25^. We transduced iRPE cells with a multiplicity of infection (MOI) of 1 or 5 and measured *RPE65* mRNA level and the lentiviral genome relative copy number by qPCR five days later (Fig. 6a). The *RPE65* mRNA level was regressed against the lentiviral genome relative copy number (Fig. 6b), revealing a substantial prediction of the mRNA level by the lentiviral genome relative copy number. The expression of *RPE65* mRNA was significantly upregulated after the gene augmentation treatment for both MOI 1 and 5 compared to untreated control (35.00 ± 21.27 and 137.34 ± 95.22 times more *RPE65* mRNA compared to control, respectively; mean ± SD; Fig. 6c). The RPE65 protein level was also investigated by fluorescence immunoblotting densitometry (Fig. 6d,e) for MOI 5-transduced iRPE and showed an upregulation that was not significant (1.55 ± 0.63-fold of Ctrl, mean ± SD).

The same experiment was repeated with two other RPE lines, namely fRPE and iCell RPE line, a stem cell-derived RPE line commercially available. Instead of 5 days, RPE cells were collected 14 days post-transduction to leave more time for the transgene to be expressed (transduction at day 28, collection at day 42).

**Figure 7:**
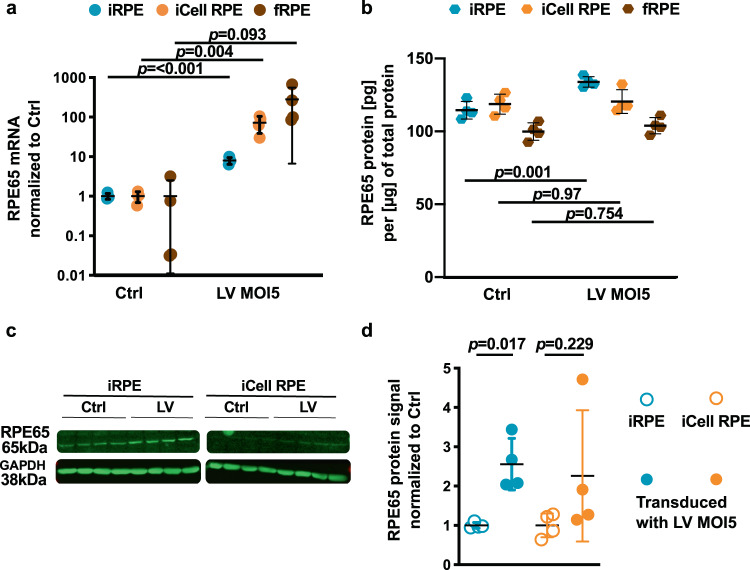
Lentivirus-mediated *RPE65* gene augmentation expression in several RPE models, but poorly affects RPE65 protein level. iRPE, iCell RPE and fRPE cells were transduced with a lentivirus expressing *RPE65* cDNA under the control of the R0.8 partial *RPE65* promoter. Relative *RPE65* mRNA and protein expressions were quantified  fourteen days later. (**a**) Comparison of normalized *RPE65* mRNA expressions between untreated controls (Ctrl) and iRPE, iCell RPE or fRPE cells transduced with R0.8-RPE65 lentivirus at MOI 5. Dunn’s multiple comparisons test after Kruskal-Wallis test. P-value significance threshold: 0.05. Data are expressed as mean ± SD. (**b**) Comparison of RPE65 protein expression between untreated controls (Ctrl) and iRPE, iCell RPE or fRPE cells transduced with R0.8-RPE65 lentivirus at MOI 5 using unpaired t-tests. P-value significance threshold: 0.05. Data are expressed as mean ± SD. (**c**) Near infra-red imaging of RPE65 and GAPDH proteins on immunoblot. (**d**) Western blot densitometry analysis of RPE65 protein level normalized to GAPDH between untreated (Ctrl) and R0.8-RPE65 lentivirus-transduced iRPE, iCell RPE and fRPE cells (MOI 5). Mean ± SD. Unpaired t-test p-value significance threshold: 0.05.

Both iCell RPE and our iRPE line transduced with the *RPE65* gene augmentation therapy presented a significant upregulation of *RPE65* mRNA expression compared to their Ctrl (71.58 ± 32.63 and 7.94 ± 1.51-fold upregulation respectively; mean ± SD, Fig. 7a). fRPE showed a high but non-significant upregulation of *RPE65* mRNA expression (280.73 ± 274.09-fold of Ctrl) probably due to its very low expression in the non-transduced Ctrl fRPE cells. While fRPE cells were at P3 in characterization experiments, they were at P5 in this experiment and their characterization at the time of transduction (Supp. Fig. 7) suggests a certain loss of RPE identity at this higher passage. Although all RPE markers tested were expressed, their expression was substantially lower than for our iRPE or the iCell RPE (Supp. Fig. 7a). In addition, immunohistochemistry (Supp. Fig. 7b) showed an absence of BEST1 protein, partial OTX2 expression and actin stress fibres that have been linked to RPE identity loss^51^.

At the protein level, only the iRPE presented an upregulation (modest) of RPE65 by ELISA (1.17 ± 0.03-fold of Ctrl, 114.5 ± 6.11 *vs* 133.9 ± 3.56 pg/µg of total protein, Fig. 7b) and Western blotting (2.55 ± 0.66-fold of Ctrl Fig. 7c,d) whereas no upregulation was observed for iCell RPE (ELISA: 1.01 ± 0.06-fold of Ctrl, 118.7 ± 6.87 *vs* 120.5 ± 8.11 pg/µg of total protein; WB: 2.26 ± 1.67-fold of Ctrl) and fRPE (ELISA: 1.04 ± 0.05-fold of Ctrl, 99.9 ± 5.98 *vs* 103.9 ± 5.6 pg/µg of total protein). Worthy of note, RPE65 protein could not be quantified in fRPE due to a weak signal out of the range of the standard curve, which is consistent with the characterization results discussed previously. Although the outcomes were consistent between ELISA and WB, no correlation in RPE65 quantification was found between the two methods (R^2^:0.167, p-value= 0.116, n = 16).

## Discussion

In this study, we modified a protocol to differentiate hiPSC into RPE and performed an extensive characterization of the resulting iRPE to validate its RPE identity. The majority of RPE marker genes investigated showed a significant upregulation after differentiation, whereas *MERTK* and *EZR*, two genes involved in the phagocytosis of POS^52,53^ did not. Several publications confirmed *MERTK* expression in ESC-derived RPE or iRPE by conventional PCR, while only a few investigated the *MERTK* expression level by qPCR. However, none compared iRPE and iPSC *MERTK* expression^14,54^. Only Liao and colleagues^55^ showed a conserved *MERTK* level after differentiation by microarray transcriptome analysis, suggesting that *MERTK* mRNA expression may play a role in pluripotent cells. Similarly, most publications investigated *EZR* protein expression by immunohistochemical staining, and none by RT-qPCR. Transcriptomic and proteomic stem cell data from repositories (respectively StemMapper^56^, and PepTracker^®^^57^) confirmed *EZR* expression in stem cells suggesting also a role of this gene in the pluripotent cells function. Although little differences of gene expression for *MERTK* and *EZR* were observed, the mRNAs levels of these genes were substantial revealing, along with the other markers, a good differentiation of the hiPSC into RPE.

*In vivo*, the RPE is responsible for recycling the 11-cis retinal chromophore through the visual cycle, in which RPE65 and LRAT proteins are involved. While *RPE65* expression was upregulated, *LRAT* exhibited a downregulation in iRPE compared to iPSCs. To our knowledge, no publication investigated *LRAT* mRNA level by qPCR in iRPE compared to iPSCs, however conventional PCR results from most studies suggest a weak *LRAT* expression in iRPE and ESC-derived RPE^5,15,58,59^. In a previous work using RT-PCR, LRAT expression was not detected in the iPSC whereas it was upregulated during RPE differentiation^60^. This different result in comparison to the present work may be related to the sensitivity of the method used and/or stem cell line used to derive RPE. Transcriptomic and proteomic data respectively from StemMapper and PepTracker^®^ confirm *LRAT* expression in stem cells. When examining the relative expression in iPSCs, it is interesting to note that *LRAT* is more expressed than *KLF4* (Supp. Fig. 1a), a result that needs confirmation in other iPSCs lines. Thus, similarly to *MERTK* and *EZR*, *LRAT* upregulation in iRPE compared to iPSCs may not be indicative of RPE functions. A functional assay investigating the chromophore recycling capability of iRPE as performed by Maeda and colleagues^59^ could confirm this hypothesis. Moreover, previous reports have shown that the LRAT protein is 25’000-fold more active^61^ than the RPE65 one, supporting the hypothesis that LRAT does not need to be highly expressed to perform its function, in contrast to RPE65. Although *LRAT* expression is positive, but weak, one cannot exclude that our culture conditions are not optimal to promote the full maturation of the iRPE. Carr and colleagues^62^ have shown that iRPE in contact with retinal explant have an increased microvilli length, suggesting an adaptation of iRPE to the culture environment.

Regarding the expression of *MITF-M*, a melanocyte-specific isoform of *MITF*, an upregulation in iRPE compared to iPSCs was observed. Previous publications showed no expression of *MITF-M* in human immortalized RPE cell lines^63–65^. However, our results suggest that *MITF-M* expression in iRPE and fRPE is possible, although at a very low level, confirming results obtained by Maruotti and colleagues^42^. In comparison, the *MITF-H* isoform that is well expressed in RPE^42,65^ is approximately 300 times more expressed than *MITF-M* in our iRPE (Supp. Fig. 1a).

Lying at the interface between the choroid and the retina, the RPE helps to maintain the homeostasis of both tissues and is therefore a highly polarized epithelium. Immunohistochemical analyses showed a polarized distribution of some RPE marker proteins, as well as a polarized ultrastructure by electron microscopy. The iRPE displayed a tendency for a polarized secretion of VEGF but not PEDF, unlike previous reports have shown^14,36,38,43^.The difference in PEDF secretion polarization with other works on iRPE and ESC-derived RPE may originate from the culture conditions. Indeed, Kamao and colleagues^38^ cultured their iRPE on type I-A collagen that contains a PEDF binding site^66,67^ which could potentially buffer PEDF and limit its secretion to the basal compartment resulting in a polarized secretion of PEDF towards the apical compartment. Our iRPE were cultured on MRF that does not contain type I but type IV collagen to which PEDF does not bind^68^. The polarized secretion of PEDF by the RPE *in vivo* may also be the result of PEDF buffering by the Bruch’s membrane, composed of collagens type I and III^69,70^, among many other proteins. However, other publications reported a polarized secretion of iRPE on substrate without type I collagen^14,43^, suggesting that the RPE inherently secretes PEDF in a polarized manner. Interestingly, Singh and colleagues^14^ observed a polarized secretion with the same culture medium used in this study suggesting that the differentiation process may be responsible for this discrepancy. Improvement of the differentiation protocol by the addition of small molecules or proteins, such as Activin A, may help to promote a better differentiation and polarization^4^. Moreover, supplementation of the culture medium with ROCK inhibitor has shown significant results on F-actin stress fibre morphology of RPE cells^51^ and could be beneficial to iRPE polarization.

Overall, the iRPE displayed many features of *bona fide* RPE. Its RPE identity was further confirmed by the transduction of lentiviral GFP reporter constructs bearing RPE specific promoters or promoters specific for other cell types. As the differentiation protocol we used promotes a neural and then retinal fate, we tested promoters of lineages that could have arisen during differentiation, namely glia (*GFAP*), retina (*RHO*), and neuronal (*SYN1*, rat *Eno2*) specific promoters. Notably, only the specific *RPE65* and ubiquitous *EFS* promoters drove GFP expression and no leakiness from aspecific promoters for RPE was observed.

Finally, we transduced iRPE cells differentiated from healthy iPSCs with an *RPE65* gene augmentation approach previously tested on *RPE65* deficiency mouse models^23,24^ and healthy non-human primates^25^. Interestingly, the *RPE65* mRNA level could be substantially increased despite the iRPE already expressing *RPE65* and the use of a partial *RPE65* promoter. However, no marked increase of the protein level was observed. This experiment was repeated with two other RPE models, namely fRPE and iCell RPE cells (commercially available stem cell-derived RPE), and with a longer transgene expression period (5 *vs* 14 days). A significant upregulation of *RPE65* mRNA expression was observed in iRPE and iCell RPE transduced cells, although the magnitude of the increase was lower in iRPE than in previous results for the same MOI (~10-fold *vs ~*130-fold). At the protein level, a significant but modest increase in RPE65 was observed by Western blotting and ELISA for the iRPE (in spite of the lower *RPE65* mRNA upregulation), but none for the iCell RPE and fRPE. The longer transgene expression period might have helped to reach a significant RPE65 protein upregulation in iRPE although the magnitude in the increase remains modest, confirming a discrepancy between *RPE65* mRNA and protein level increase. In total, three different batches of lentivirus were used (two for Fig. 6 and one for Fig. 7) which may explain the heterogeneity observed in *RPE65* mRNA level expression post-transduction in iRPE. In addition, heterogeneity between iRPE batches may also be involved, although their characterization showed consistent results.

The difference of magnitude between the mRNA and protein upregulation in all 3 RPE models suggests a potential post-transcriptional regulation mechanism that has yet to be elucidated. Although inhibitory translational regulation of the *RPE65* mRNA by miRNA has been described^71,72^, this regulation was mediated by the 3’UTR that is absent from the lentiviral construct containing only the *RPE65* coding sequence. To our knowledge, no other publication reported post-transcriptional regulation mechanisms of *RPE65*. This mechanism of regulation may be of great interest for gene augmentation therapy since it suggests no potential toxic effects due to high gene doses. Nonetheless, such observations need to be confirmed in RPE deficient for *RPE65* or expressing mutated forms of the protein.

Several clinical trials have been conducted to treat LCA2 caused by *RPE65* deficiency^73–75^, one of which led to the first AAV-based gene therapy to reach the market^76^. Although AAV-mediated *RPE65* gene augmentation therapy showed substantial vision improvements in the patients, none proved to prevent the progression of the photoreceptor degeneration observed^77^, contrasting with LCA2 animal model results^23,78^. This discrepancy may originate from the different levels of RPE65 protein needed to perform physiological functions between animals and human beings^22^, which suggests that a higher concentration of RPE65 is necessary to provide an optimal rescue in human. Bainbridge and colleagues^74^ observed a limiting dose of 10^12^ vector genomes inducing no toxicity, therefore precluding from using higher amounts to achieve higher RPE65 protein expression, and creating the need for a more potent gene therapy. Georgiadis and colleagues^79^ developed an AAV-based optimized RPE65 (OPTIRPE65) gene augmentation therapy 300 times more potent than their original work and were able to reach a plateau of rescue with only 10^9^ vector genomes in the *Rpe65* deficiency mouse model. Interestingly, when they tested their OPTIRPE65 on wild-type mice they observed physiological expression of the *RPE65* transgene, meaning a doubling of the overall cDNA *Rpe65* expression. Although comparison with our *in vitro* results is tricky due to differences in vector doses, vector biodistribution, model (endogenous RPE *vs* iPSC-derived) and methodology (absolute *vs* relative mRNA quantification), the significant increase in *RPE65* mRNA level (up to 135-fold at MOI 5) suggests a high potential of the LV-based gene therapy. Further studies on the *RPE65-*deficient iRPE model are needed to confirm that this strategy is able to rescue *RPE65* mRNA and protein to a physiological level, and which vector dose is needed to attain such concentration.

Lentiviral vectors have long been used in gene therapy, but their use has mostly been restricted to *ex-vivo* gene therapy in human^80^. Studies of lentiviral integration revealed that more than 60% of the insertions occur in transcription regions, but rarely in promoter sequences^81,82^, reducing the risk of tumorigenesis as the disruption of a promoter sequence generally impacts more genes than the disruption of a transcription region. Moreover, RPE is a post-mitotic tissue and adenomas of the RPE are seldom while adenocarcinomas are even rarer^83^, suggesting the RPE is a safer target for LV integration than most tissues. In addition to safety studies in non-human primate^25^, studying the safety of lentiviral-mediated gene therapy in *in vitro* models such as iRPE derived from RPE65 deficient patient or engineered by CRISPR/Cas9 to knockdown the *RPE65* gene is an important preclinical step to elucidate untoward effect occurring in this cell type before considering its translation to clinic.

### Conclusions and future directions

In conclusion, we modified an RPE differentiation protocol that produces iRPE bearing close features to *bona fide* RPE. The efficiency of this differentiation protocol should still be validated in other hiPSC lines. The iRPE revealed to be a suitable *in vitro* model for lentiviral gene therapy tests after transduction of several different constructs, confirming the lentivirus tropism for RPE previously observed *in vivo*. *RPE65* gene augmentation therapy in iRPE cells resulted in high *RPE65* mRNA expression corroborating previous results obtained in animal models^23,24^ but only elicited a modest RPE65 protein increase. Future directions include the production of an *RPE65*-deficient iRPE model to further test the lentiviral vector gene therapy and demonstrate its safety and efficacy in human cells.

## Methods

### RPE differentiation protocol and iRPE culture

A detailed protocol is available in Supplementary Methods (SM). Briefly, hiPSCs described in Singh *et al*.^14^ were cultured in feeder-free conditions in either mTeSR-1 or E8 media kit (StemCell) on Matrigel Embryonic Stem Cell grade (Corning). hiPSC clumps (between P18 and P30) were lifted using type IV collagenase (LifeTechnologies) and cultured overnight in free floating conditions in Embryoid Body Medium (EBM, see SM for composition). Resulting Embryoid Body-like aggregates (EBs) were plated on Matrigel Reduced Factor (MRF, Corning) in a neural induction medium (NIM also described in SM) changed every 3 days. On day 10, medium was switched to a Retinal Differentiation Medium with vitamin A (RDMcA). On day 13, medium was switched to a version without vitamin A (RDMsA). Pigmented foci (PF) typically appeared between days 18 and 24 and matured until collection at day 30. PF were plated on MRF as a purification and expansion step. Resulting pigmented patches were collected after 20 to 30 days, dissociated and plated on MRF at a density of 0.5 to 1·10^5^ cells·cm^−2^ (P1 stage) to obtain pure RPE cell culture. NIM, RDMcA and RDMsA are adapted from^6^. Passage 3 iRPE cells at 42 days in culture on MRF-coated transwell (CLS3460, Sigma-Aldrich) were used for all assays of this publication unless otherwise stated. Images from Fig. 1(b–e) depicting the differentiation process were taken with a DMS300 microscope from Leica.

### fRPE and iCell RPE culture

Human foetal retinal pigment epithelium (fRPE) cells were acquired from Lonza (Ref. 00194987, lot number 0000465423) and cultured according to the manufacturer’s protocol in RtEGM^®^Bulletkit^®^ (Ref. 00195409, Lonza). Human iCell induced pluripotent stem cell-derived RPE (iCell RPE) cells were acquired from StemCell (Line 01279, Ref. C1047, lot103261) and cultured in RDMsA. For any assay performed on fRPE or iCell RPE, the cells were used at P3 and grown on MRF-coated transwells (CLS3460, Sigma-Aldrich) for 42 days in RDMsA (unless stated otherwise). All tests were performed with the same fRPE or iCell RPE line and, for fRPE, several maturation tests were done independently.

### RNA extraction and analysis of gene expression

RNA isolation was performed using TRI Reagent^®^ (T9424, Sigma-Aldrich) according to the manufacturer’s protocol. Retro-transcription PCR was performed using the High Capacity cDNA Reverse Transcription Kit from Applied Biosystems (Ref. 4368814) according to manufacturer’s protocol with a Biometra Tadvanced thermocycler from Analytik Jena AG. For each RNA sample, a negative control lacking the retrotranscriptase in the retrotranscription step was used in qPCR. Gene expression was assessed by qPCR using the LightCycler^®^96 and the FastStart Essential DNA Green Master mix (Ref. 06402712001) from Roche. Experimental design and gene expression quantification are explained in SM. *ATP5F1B*, *SRP72* and *GPI* reference genes were chosen after having reviewed literature^42,84^ and tested their stability in iPS, iRPE, fRPE and iCell RPE. Primer sequences, primer efficiencies, qPCR programmes and additional information on qPCR data processing are available in SM.

### Immunohistochemistry and imaging

iRPE, fRPE and iCell RPE cells were cultured on 12 mm diameter transwells, (CLS3460, Sigma-Aldrich) coated with MRF (at a 1:30 dilution) in RDMsA for 42 days (unless stated otherwise) before processing for immunohistochemistry (see SM for detailed protocol). iPS on coverslips were processed for immunohistochemistry at around 60% confluence. Briefly, cells were fixed in 4% paraformaldehyde for 10-20 min at room temperature (RT), rinsed with PBS and blocked for 3-5 h in 5% foetal bovine serum + 5% normal goat serum + 0.1% Triton X-100 in PBS 1×. Primary antibody was incubated overnight at 4 °C in blocking solution (see SM for antibody references and dilutions). Primary antibody was washed with PBS 1X before secondary antibody incubation (see SM) 1 h at RT in PBS 1×. After wash with PBS 1×, the transwell membrane was cut and mounted in Mowiol. Z-stacks were acquired using a LSM700 confocal microscope from Zeiss at the EPFL BioImaging & Optics Core Facility (EPFL-BIOP). Other images were acquired with a BX60 microscope from Olympus equipped with a DP72 camera or a Leica DM6B microscope equipped with a DFC9000GT camera.

### Electron microscopy

iRPE cells cultured on 6.5 mm transwells (CLS3470, Sigma-Aldrich) for 45 days were washed with PBS 1×, fixed in 2.5% glutaraldehyde for 90 min and left in PBS 1X until processing and imaging by the Electron Microscopy Facility of the University of Lausanne (EMF-UNIL). Details of processing and acquisition are available in SM.

### Transepithelial resistance measurements

Transepithelial resistance measurements were performed with an EVOM2 Epithelial Voltohmmeter (World Precision Instrument) on iRPE and fRPE cells grown on 12 mm diameter transwells. One transwell with only MRF coating was used as control. Transepithelial resistance was determined as the mean of three iRPE/fRPE transwell resistance measurements minus the control resistance times the transwell surface (1.12 cm^2^).

### POS isolation and phagocytosis assay

Photoreceptor outer segments (POS) isolation and phagocytosis assay protocol were adapted from^46^ (see SM for detailed protocol). Briefly, fresh bovine eyes were collected from a local slaughterhouse and dissected in dim red light. Retinas were collected in a pre-chilled homogenization Tris buffer-based solution and ultracentrifugation was performed in a sucrose gradient (113’000 g during 48 min at 4 °C). The resulting pink band - characteristic of rhodopsin - was collected and washed three times. POS concentration was determined using a Neubauer chamber and aliquots of 5·10^6^ POS were stored at -80 °C until use. Thawed POS were incubated with an FITC solution to obtain FITC-conjugated POS and fed to iRPE or fRPE cells for 16 hours (5·10^6^ POS/transwell). iRPE or fRPE cells were washed to remove the non-internalized and unbound POS, immunolabelled for rhodopsin without the permeabilization step, and fixed in 4% paraformaldehyde before mounting on microscope slides for imaging.

### PEDF and VEGF quantifications

Pigment epithelium-derived factor (PEDF) and vascular endothelial growth factor (VEGF) concentrations in medium were quantified using ELISA kits (RD191114200R from Biovendor, Brno, Czech Republic and RAB0507 from Sigma-Aldrich, respectively). iRPE cells cultured on MRF-coated transwells for 42 days were rinsed with HBSS twice and fresh RDMsA was added. Twenty-four hours later, the RDMsA was collected from the apical and basal chambers and stored at -80 °C. ELISAs were performed maximum one week after sample collection. For each iRPE cell line tested, triplicate samples (i.e. iRPE cell wells) were quantified in ELISA technical duplicates (i.e. ELISA wells). Mean of triplicate samples was calculated and Wilcoxon match-pairs signed-rank tests comparing contents of basal and apical chambers for VEGF and PEDF were performed using GraphPad Prism 6 software.

### Lentiviral vector production and titration

Lentiviral vector and titration protocols have already been described in^23^, however detailed protocols are available in SM. Briefly, HEK293T cells were transiently transfected with a second generation lentiviral packaging system, culture supernatants were collected two days later and underwent two rounds of ultracentrifugation (70’000 g, 90 min, 4 °C) to wash and pellet the lentivirus that was resuspended, aliquoted and stored at -80 °C until use.

### Lentiviral transduction of iRPE, iCell RPE and fRPE cells with GFP constructs driven by different promoters

The 6 different lentiviral constructs were originally designed in the following publications: hLox-Ro.8-GFP-WPRE^23^, hLox-EFS-GFP-WPRE^85^, hLox-GFAP-GFP-WPRE (Unpublished – details in SM), hLox-Rhodopsin-GFP-WPRE^85^, SIN-ratNSE-AcGFPnuc-WPRE^86^, and SIN-Synapsin-GFP-WPRE (Unpublished – details in SM). iRPE and fRPE cells were cultured on transwells for 42 days and transduced with one of the aforementioned lentiviral vectors at a multiplicity of infection (MOI) of 1. Five days later, iRPE and fRPE cells were collected, fixed and one part of the cells were analyzed at the Flow Cytometry Facility of the University of Lausanne (on a BD FACSCalibur^®^ cell analyser, BD Biosciences) to determine the proportion of GFP-positive cells while genomic DNA (gDNA) was extracted from the other part to quantify lentiviral genome relative copy number by qPCR (details available in SM).

### Lentiviral-mediated RPE65 augmentation therapy

The Good Manufacturing Practices (GMP)-compliant construct pCCL-R0.8-RPE65-WPRE4^25^ was used to produce lentivirus delivering the RPE65 protein cDNA under the control of the partial *RPE65* promoter R0.8. RPE cells were transduced with this lentivirus at targeted MOI of 1 and 5. Five (Fig. 6) or 14 (Fig. 7) days later, RPE cells were collected, RNA and gDNA extracted using TRI reagent^®^ and protein isolated using a custom lysis buffer (details available in SM). *RPE65* mRNA expression and lentiviral genome relative copy number were determined by qPCR (details available in SM).

RPE65 protein level was determined by an RPE65 ELISA kit according to the manufacturer’s protocol (XPEH2516, XpressBio, see SM for details) or Western blotting. For Western blotting, 40 to 50 µg total protein lysates were run on a 10% acrylamide gel, transferred to a PVDF membrane, the membrane was then blocked in Odyssey^®^ TBS Blocking Buffer (P/N 927-50100, Licor), incubated overnight at RT with RPE65 and α-Actin or GAPDH primary antibodies, washed in TBS containing 0.1% Tween-20, incubated with near infra-red (680 nm and 800 nm emitting) conjugated fluorophore secondary antibodies and washed again. Blot imaging was performed with an Azure c600 imaging system and densitometry analysis was performed with the Image Studio Lite software from Licor. Detailed Western blotting procedure is available in SM as well as original blots in Supp. Fig. 6.

### Statistical analysis

All statistical analyses were performed and graphs realized with Graphpad Prism 6 software. The number of biological replicates is stated on figures and in their caption, as well as statistical tests used and the p-value significance level. For the characterization experiments, one biological replicate corresponds to one iRPE line differentiated from hiPSCs. For *RPE65* gene augmentation therapy experiments, one biological replicate corresponds to one transwell.

## Supplementary information

Supplementary Information.

## Data Availability

The datasets generated and/or analysed during the current study are available from the corresponding author upon reasonable request.

## References

[1] Strauss O (2005). The Retinal Pigment Epithelium in Visual Function. Physiol. Rev..

[2] den Hollander AI, Roepman R, Koenekoop RK, Cremers FPM (2008). Leber congenital amaurosis: Genes, proteins and disease mechanisms. Prog. Retin. Eye Res..

[3] Hartong DT, Berson EL, Dryja TP (2006). Retinitis pigmentosa Prevalence and inheritance patterns. Lancet.

[4] Leach LL, Clegg DO (2015). Concise Review: Making Stem Cells Retinal: Methods for Deriving Retinal Pigment Epithelium and Implications for Patients with Ocular Disease. Stem Cells.

[5] Vugler A (2008). Elucidating the phenomenon of HESC-derived RPE: Anatomy of cell genesis, expansion and retinal transplantation. Exp. Neurol..

[6] Meyer JS (2009). Modeling early retinal development with human embryonic and induced pluripotent stem cells. Proc. Natl. Acad. Sci..

[7] Haruta M (2004). *In vitro* and *in vivo* characterization of pigment epithelial cells differentiated from primate embryonic stem cells. Investig. Ophthalmol. Vis. Sci.

[8] Sohn EH (2015). Allogenic iPSC-derived RPE cell transplants induce immune response in pigs: A pilot study. Sci. Rep.

[9] Schwartz SD (2012). Embryonic stem cell trials for macular degeneration: A preliminary report. Lancet.

[10] Song WK (2015). Treatment of macular degeneration using embryonic stem cell-derived retinal pigment epithelium: Preliminary results in Asian patients. Stem Cell Reports.

[11] Mandai M (2017). Autologous Induced Stem-Cell–Derived Retinal Cells for Macular Degeneration. N. Engl. J. Med..

[12] Da Cruz L (2018). Phase 1 clinical study of an embryonic stem cell-derived retinal pigment epithelium patch in age-related macular degeneration. Nat. Biotechnol..

[13] Schwartz SD (2015). Human embryonic stem cell-derived retinal pigment epithelium in patients with age-related macular degeneration and Stargardt’s macular dystrophy: Follow-up of two open-label phase 1/2 studies. Lancet.

[14] Singh R (2013). Functional analysis of serially expanded human iPS cell-derived RPE cultures. Investig. Ophthalmol. Vis. Sci.

[15] Cereso N (2014). Proof of concept for AAV2/5-mediated gene therapy in iPSC-derived retinal pigment epithelium of a choroideremia patient SI. Mol. Ther. - Methods Clin. Dev.

[16] Galloway CA (2017). Drusen in patient-derived hiPSC-RPE models of macular dystrophies. Proc. Natl. Acad. Sci. U. S. A..

[17] Pennesi ME, Neuringer M, Courtney RJ (2012). Animal models of age related macular degeneration. Mol. Aspects Med..

[18] Li Y (2014). Gene therapy in patient-specific stem cell lines and a preclinical model of retinitis pigmentosa with membrane frizzled-related protein defects. Mol. Ther..

[19] Le Meur G (2007). Restoration of vision in RPE65-deficient Briard dogs using an AAV serotype 4 vector that specifically targets the retinal pigmented epithelium. Gene Ther.

[20] Acland GM (2005). Long-term restoration of rod and cone vision by single dose rAAV-mediated gene transfer to the retina in a canine model of childhood blindness. Mol. Ther..

[21] Annear JM (2013). Successful Gene Therapy in Older Rpe65-Deficient Dogs. Hum. Gene Ther..

[22] Bainbridge JWB (2015). Long-Term Effect of Gene Therapy on Leber’s Congenital Amaurosis. N. Engl. J. Med..

[23] Bemelmans AP (2006). Lentiviral gene transfer of Rpe65 rescues survival and function of cones in a mouse model of leber congenital amaurosis. Plos Med..

[24] Kostic Corinne, Crippa Sylvain Vincent, Pignat Vérène, Bemelmans Alexis-Pierre, Samardzija Marijana, Grimm Christian, Wenzel Andreas, Arsenijevic Yvan (2011). Gene Therapy Regenerates Protein Expression in Cone Photoreceptors in Rpe65R91W/R91W Mice. PLoS ONE.

[25] Matet A (2017). Evaluation of tolerance to lentiviral LV-RPE65 gene therapy vector after subretinal delivery in non-human primates. Transl. Res..

[26] Nieto-Estévez V, Defterali Ç, Vicario-Abejón C (2016). IGF-I: A key growth factor that regulates neurogenesis and synaptogenesis from embryonic to adult stages of the brain. Front. Neurosci.

[27] Versteyhe S (2013). IGF-I, IGF-II, and insulin stimulate different gene expression responses through binding to the IGF-I receptor. Front. Endocrinol. (Lausanne).

[28] Duester G (2009). Keeping an eye on retinoic acid signaling during eye development. Chem. Biol. Interact..

[29] Harpavat S, Cepko CL (2003). Thyroid Hormone and Retinal Development: An Emerging Field. Thyroid.

[30] Cvekl A, Wang WL (2009). Retinoic acid signaling in mammalian eye development. Exp. Eye Res..

[31] Martínez-Morales JR, Rodrigo I, Bovolenta P (2004). Eye development: A view from the retina pigmented epithelium. BioEssays.

[32] Klimanskaya I (2004). Derivation and Comparative Assessment of Retinal Pigment Epithelium from Human Embryonic Stem Cells Using Transcriptomics. Cloning Stem Cells.

[33] Idelson M (2009). Directed Differentiation of Human Embryonic Stem Cells into Functional Retinal Pigment Epithelium Cells. Cell Stem Cell.

[34] Saari JC (2012). Vitamin A Metabolism in Rod and Cone Visual Cycles. Annu. Rev. Nutr..

[35] Feeney, L. Lipofuscin and melanin of human RPE. **17**, 1–9 (1978). 669890

[36] Zhu D (2011). Polarized secretion of PEDF from human embryonic stem cell-derived RPE promotes retinal progenitor cell survival. Investig. Ophthalmol. Vis. Sci.

[37] Zhu Yu, Carido Madalena, Meinhardt Andrea, Kurth Thomas, Karl Mike O., Ader Marius, Tanaka Elly M. (2013). Three-Dimensional Neuroepithelial Culture from Human Embryonic Stem Cells and Its Use for Quantitative Conversion to Retinal Pigment Epithelium. PLoS ONE.

[38] Kamao H (2014). Characterization of human induced pluripotent stem cell-derived retinal pigment epithelium cell sheets aiming for clinical application. Stem Cell Reports.

[39] Sonoda S (2009). A protocol for the culture and differentiation of highly polarized human retinal pigment epithelial cells. Nat. Protoc..

[40] Blenkinsop TA, Salero E, Stern JH, Temple S (2012). The Culture and Maintenance of Functional Retinal Pigment Epithelial Monolayers from Adult Human Eye. In Journal of the American Medical Association.

[41] Kokkinaki M, Sahibzada N, Golestaneh N (2011). Human induced pluripotent stem-derived retinal pigment epithelium (RPE) cells exhibit ion transport, membrane potential, polarized vascular endothelial growth factor secretion, and gene expression pattern similar to native RPE. Stem Cells.

[42] Maruotti J (2013). A Simple and Scalable Process for the Differentiation of Retinal Pigment Epithelium From Human Pluripotent Stem Cells. Stem Cells Transl. Med.

[43] Plaza Reyes A (2016). Xeno-Free and Defined Human Embryonic Stem Cell-Derived Retinal Pigment Epithelial Cells Functionally Integrate in a Large-Eyed Preclinical Model. Stem Cell Reports.

[44] Bhutto IA (2006). Pigment epithelium-derived factor (PEDF) and vascular endothelial growth factor (VEGF) in aged human choroid and eyes with age-related macular degeneration. Exp. Eye Res..

[45] Bosch E, Horwitz J, Bok D (1993). Phagocytosis of Outer Segments by Retinal Pigment Epithelium: Phagosome-Lysosome Interaction. J. Histochem. Cytochem..

[46] Mao Y, Finnemann SC (2013). Analysis of photoreceptor outer segment phagocytosis by RPE cells in culture. Methods Mol. Biol.

[47] Miyoshi H, Takahashi M, Gage FH, Verma IM (1997). Stable and efficient gene transfer into the retina using an HIV-based lentiviral vector. Proc. Natl. Acad. Sci. U. S. A..

[48] Bemelmans AP (2005). Retinal cell type expression specificity of HIV-1-derived gene transfer vectors upon subretinal injection in the adult rat: Influence of pseudotyping and promoter. J. Gene Med..

[49] Hashimoto T (2007). Lentiviral gene replacement therapy of retinas in a mouse model for Usher syndrome type 1B. Gene Ther.

[50] Holmgaard A (2017). *In Vivo* Knockout of the Vegfa Gene by Lentiviral Delivery of CRISPR/Cas9 in Mouse Retinal Pigment Epithelium Cells. Mol. Ther. - Nucleic Acids.

[51] Müller C, Charniga C, Temple S, Finnemann SC (2018). Quantified F-Actin Morphology Is Predictive of Phagocytic Capacity of Stem Cell-Derived Retinal Pigment Epithelium. Stem Cell Reports.

[52] Nandrot E (2000). Homozygous deletion in the coding sequence of the c-mer gene in RCS rats unravels general mechanisms of physiological cell adhesion and apoptosis. Neurobiol. Dis..

[53] Finnemann, S. C. & Nandrot, E. F. Mertk Activation During RPE Phagocytosis *in Vivo* Requires αVβ5 Integrin. In *Advanced Experimentl Medical Biology* **572**, 499–503 (Springer US, 2006). 10.1007/0-387-32442-9_69 PMC3577060 17249615

[54] Reichman S (2014). From confluent human iPS cells to self-forming neural retina and retinal pigmented epithelium. Proc. Natl. Acad. Sci..

[55] Liao JL (2010). Molecular signature of primary retinal pigment epithelium and stem-cell-derived RPE cells. Hum. Mol. Genet.

[56] Pinto JP (2018). StemMapper: A curated gene expression database for stem cell lineage analysis. Nucleic Acids Res.

[57] Brenes A, Afzal V, Kent R, Lamond AI (2018). The Encyclopedia of Proteome Dynamics: A big data ecosystem for (prote)omics. Nucleic Acids Res.

[58] Carr Amanda-Jayne, Vugler Anthony A., Hikita Sherry T., Lawrence Jean M., Gias Carlos, Chen Li Li, Buchholz David E., Ahmado Ahmad, Semo Ma'ayan, Smart Matthew J. K., Hasan Shazeen, da Cruz Lyndon, Johnson Lincoln V., Clegg Dennis O., Coffey Pete J. (2009). Protective Effects of Human iPS-Derived Retinal Pigment Epithelium Cell Transplantation in the Retinal Dystrophic Rat. PLoS ONE.

[59] Maeda A, Palczewski K (2013). Retinal degeneration in animal models with a defective visual cycle. Drug Discov. Today Dis. Model.

[60] Maeda T (2013). Retinal Pigmented Epithelial Cells Obtained from Human Induced Pluripotent Stem Cells Possess Functional Visual Cycle Enzymes *in Vitro* and *in Vivo*. J. Biol. Chem..

[61] Jin M, Li S, Moghrabi WN, Sun H, Travis GH (2005). Rpe65 is the retinoid isomerase in bovine retinal pigment epithelium. Cell.

[62] Carr A-J (2009). Molecular characterization and functional analysis of phagocytosis by human embryonic stem cell-derived RPE cells using a novel human retinal assay. Mol. Vis..

[63] Amae S (1998). Identification of a novel isoform of microphthalmia-associated transcription factor that is enriched in retinal pigment epithelium. Biochem. Biophys. Res. Commun..

[64] Takeda K (2002). Mitf-D, a newly identified isoform, expressed in the retinal pigment epithelium and monocyte-lineage cells affected by Mitf mutations. Biochim. Biophys. Acta - Gene Struct. Expr.

[65] Hershey CL, Fisher DE (2005). Genomic analysis of the Microphthalmia locus and identification of the MITF-J/Mitf-J isoform. Gene.

[66] Meyer C, Notari L, Becerra SP (2002). Mapping the type I collagen-binding site on pigment epithelium-derived factor: Implications for its antiangiogenic activity. J. Biol. Chem..

[67] Sekiya A, Okano-Kosugi H, Yamazaki CM, Koide T (2011). Pigment epithelium-derived factor (PEDF) shares binding sites in collagen with heparin/heparan sulfate proteoglycans. J. Biol. Chem..

[68] Kozaki KI (1998). Isolation, purification, and characterization of a collagen-associated serpin, caspin, produced by murine colon adenocarcinoma cells. J. Biol. Chem..

[69] Karwatowski WSS (1995). Preparation of Bruch’s membrane and analysis of the age-related changes in the structural collagens. Br. J. Ophthalmol..

[70] Booij JC, Baas DC, Beisekeeva J, Gorgels TGMF, Bergen AAB (2010). The dynamic nature of Bruch’s membrane. Prog. Retin. Eye Res..

[71] Liu SY, Redmond TM (1998). Role of the 3′-untranslated region of RPE65 mRNA in the translational regulation of the RPE65 gene: Identification of a specific translation inhibitory element. Arch. Biochem. Biophys..

[72] Samuel W (2013). Translational Regulation of RPE65 Expression by microRNA. Invest Ophthalmol Vis Sci.

[73] Jacobson SG (2006). Safety of Recombinant Adeno-Associated Virus Type 2-RPE65 Vector Delivered by Ocular Subretinal Injection. Mol. Ther..

[74] Bainbridge JWB (2008). Effect of gene therapy on visual function in Leber’s congenital amaurosis. N. Engl. J. Med..

[75] Maguire AM (2009). Age-dependent effects of RPE65 gene therapy for Leber’s congenital amaurosis: a phase 1 dose-escalation trial. Lancet.

[76] Smalley E (2017). First AAV gene therapy poised for landmark approval. Nat. Biotechnol..

[77] Cideciyan AV (2013). Human retinal gene therapy for Leber congenital amaurosis shows advancing retinal degeneration despite enduring visual improvement. Proc. Natl. Acad. Sci..

[78] Mowat FM (2013). RPE65 gene therapy slows cone loss in Rpe65-deficient dogs. Gene Ther.

[79] Georgiadis A (2016). Development of an optimized AAV2/5 gene therapy vector for Leber congenital amaurosis owing to defects in RPE65. Gene Ther.

[80] Naldini L (2011). *Ex vivo* gene transfer and correction for cell-based therapies. Nat. Rev. Genet..

[81] Berry, C., Hannenhalli, S., Leipzig, J. & Bushman, F. D. Selection of Target Sites for Mobile DNA Integration in the Human Genome. *PLoS Comput*. *Biol*. **2** (2006). 10.1371/journal.pcbi.0020157 PMC1664696 17166054

[82] Ronen K (2009). Distribution of Lentiviral Vector Integration Sites in Mice Following Therapeutic Gene Transfer to Treat β -thalassemia. Mol. Ther..

[83] Shields J, Shields C, Gündüz K, Eagle R (1999). Neoplasms of the Retinal Pigment Epithelium. Arch. Ophthalmol..

[84] Synnergren J (2007). Differentiating Human Embryonic Stem Cells Express a Unique. Stem Cells.

[85] Kostic C (2003). Activity analysis of housekeeping promoters using self-inactivating lentiviral vector delivery into the mouse retina. Gene Ther.

[86] Delzor A (2012). Restricted transgene expression in the brain with cell-type specific neuronal promoters. Hum. Gene Ther. Methods.

