# Supplementary material for: Lentiviral mediated RPE65 gene transfer in healthy hiPSCs-derived retinal pigment epithelial cells markedly increased RPE65 mRNA, but modestly protein level
Source: Sci Rep. 2020 Jun 1;10:8890. doi: 10.1038/s41598-020-65657-y (PMC7264209; doi:10.1038/s41598-020-65657-y)
Supplement: Supplementary file 1 — Supplementary Information. [file 41598_2020_65657_MOESM1_ESM.pdf]

Supplementary Method

Lentiviral mediated *RPE65* gene transfer in healthy hiPSCs-derived retinal pigment epithelial cells markedly increased *RPE65 mRNA*, but modestly protein level.

Florian Udry[1], Sarah Decembrini[1,4], David M. Gamm[2], Nicole Déglon[3], Corinne Kostic[1], and Yvan Arsenijevic[1]*

[1]Department of ophthalmology, Unit of Retinal Degeneration and Regeneration, University of Lausanne, Hôpital ophtalmique Jules-Gonin, 1004 Lausanne, Switzerland; [2] McPherson Eye Research Institute, Waisman Center and Department of Ophthalmology and Visual Sciences, and University of Wisconsin-Madison, Madison, USA; [3], Neuroscience Research Center, Laboratory of Neurotherapies and Neuromodulation, Lausanne University Hospital and University of Lausanne, Switzerland; [4]Department of Biomedicine, University Hospital Basel & University Basel, Hebelstr. 20, 4031, Basel, Switzerland.

Keywords: RPE, iPSC, gene therapy, lentiviral vector, retinal degeneration, LCA2

*Corresponding author: Yvan Arsenijevic

Hôpital ophtalmique Jules-Gonin, avenue de France 15, 1004 Lausanne, Switzerland.

Tel: +41 (0)21 626 82 60. Email: yvan.arsenijevic@fa2.ch

Table of contents

SUPPLEMENTARY FIGURE 1 4
SUPPLEMENTARY FIGURE 2 5
SUPPLEMENTARY FIGURE 3 6
SUPPLEMENTARY FIGURE 4 8
SUPPLEMENTARY FIGURE 5 9
SUPPLEMENTARY FIGURE 6 11
SUPPLEMENTARY FIGURE 7 13

SUPPLEMENTARY METHOD 13

1. IRPE DIFFERENTIATION PROTOCOL 13
2. RNA AND DNA EXTRACTION 15
2.1 PRELIMINARY INFORMATION ON THE PROTOCOLS USED 16
2.2 GDNA EXTRACTION 16
2.3 RNA/DNA PRECIPITATION 17
3. QUANTITATIVE POLYMERASE CHAIN REACTION 17
3.1 PROGRAMS 17
3.2 PRIMER LIST 18
3.3 QPCR EFFICIENCIES 19
3.4 QPCR EXPERIMENTAL DESIGN AND QUANTIFICATION METHOD 20
4. IMMUNOHISTOCHEMISTRY 22
4.1 IMMUNOSTAINING PROTOCOL 22
4.2 VIOLIN PLOTS OF IMMUNOSTAINED PROTEIN SUBCELLULAR LOCALIZATION 23
5. ELECTRON MICROSCOPY 23
6. TER MEASUREMENTS 24
7. POS ISOLATION AND PHAGOCYTOSIS ASSAY 24
7.1 STOCK SOLUTIONS 24
7.2 WORKING SOLUTIONS 25
7.3 POS ISOLATION PROCESS 26
7.4 PHAGOCYTOSIS ASSAY 29
7.5 RHODOPSIN STAINING 29
8. LENTIVIRAL PRODUCTION AND TITRATION 30
8.1 PRODUCTION 30
8.2 TITRATION 32
8.2.1 FACS OR QPCR TITRATION 32
9. WESTERN BLOT 33
9.1 PROTEIN EXTRACTION AND QUANTIFICATION 33
9.2 GEL ELECTROPHORESIS AND PROTEIN TRANSFER 34
9.3 BLOT STAINING AND IMAGING 35
9.4 RPE65 PROTEIN QUANTIFICATION 36
10. ELISA 36

REFERENCES 36

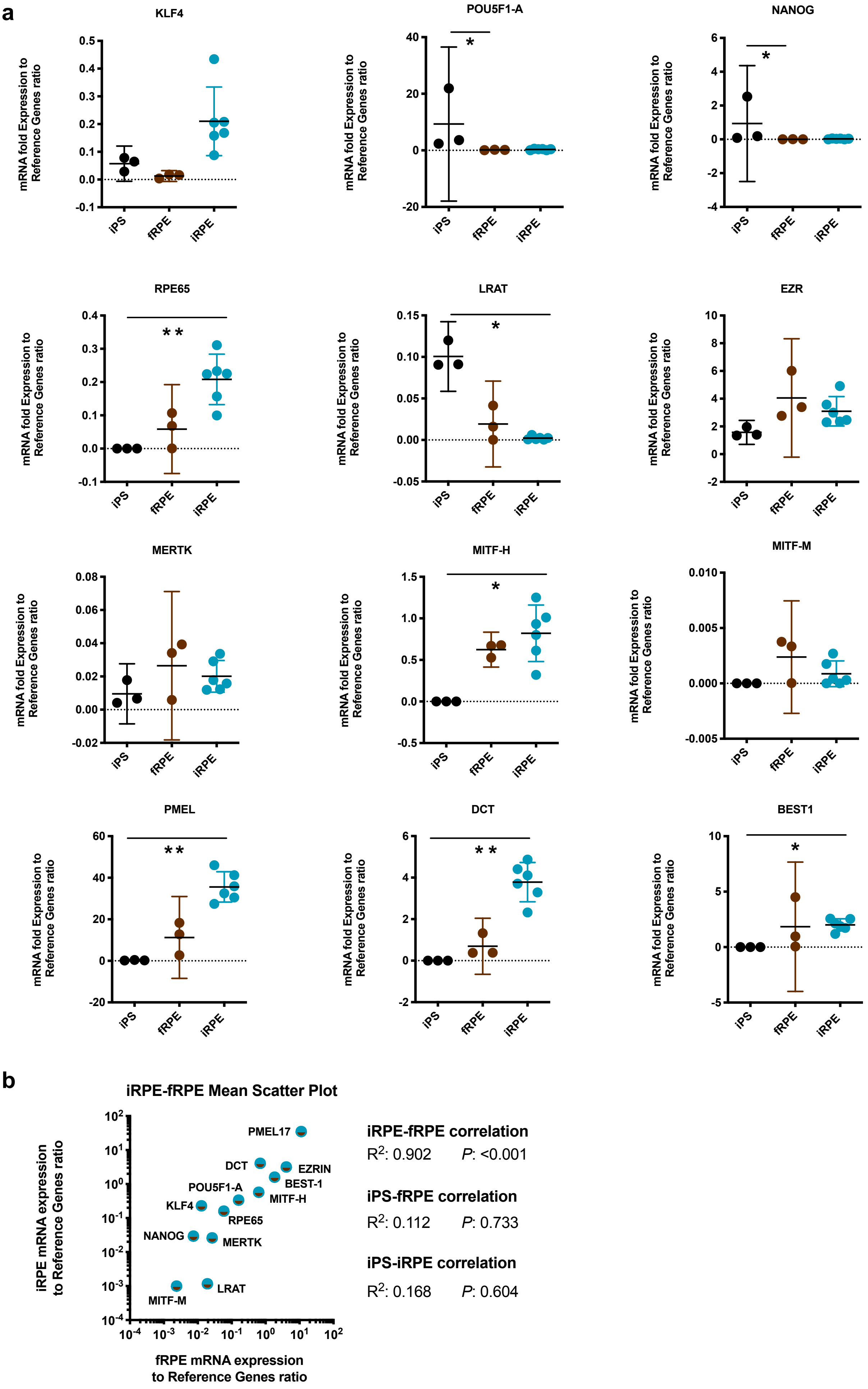

a
KLF4
POU5F1-A
NANOG
RPE65
LRAT
EZR
MERTK
MITF-H
MITF-M
PMEL
DCT
BEST1
mRNA fold Expression to Reference Genes ratio
iPS
fRPE
iRPE
*
**
b
iRPE-fRPE Mean Scatter Plot
iRPE mRNA expression to Reference Genes ratio
fRPE mRNA expression to Reference Genes ratio
PMEL17
DCT
EZRIN
BEST-1
POU5F1-A
MITF-H
KLF4
RPE65
NANOG
MERTK
LRAT
MITF-M
iRPE-fRPE correlation
R2: 0.902 P: <0.001
iPS-fRPE correlation
R2: 0.112 P: 0.733
iPS-iRPE correlation
R2: 0.168 P: 0.604

Supplementary Figure 1

RPE marker mRNA expression in iPS, fRPE and iRPE. Data are the same as in Fig. 1a but represented differently to appreciate the RPE marker genes expression normalized to the reference genes ratio. (a) Quantitative PCR investigating RPE marker expression in iPS (n=3), fRPE (n=3) and iRPE (n=6). Bars represent mean ± 95 % CI. Kruskal-Wallis tests were performed (iPS vs. iRPE and iPS vs. fRPE for each gene) followed by Dunn’s multiple comparisons test. Significant comparisons are signalled by *. (b) Scatter plot of the mean RPE marker mRNA expression (normalized to the reference genes ratio) for each gene for iRPE and fRPE. Pearson correlation tests (0.05 significance threshold) were performed between iPS vs. fRPE, iPS vs. iRPE, and iRPE vs. fRPE. R: correlation coefficients. *P*: p-values.

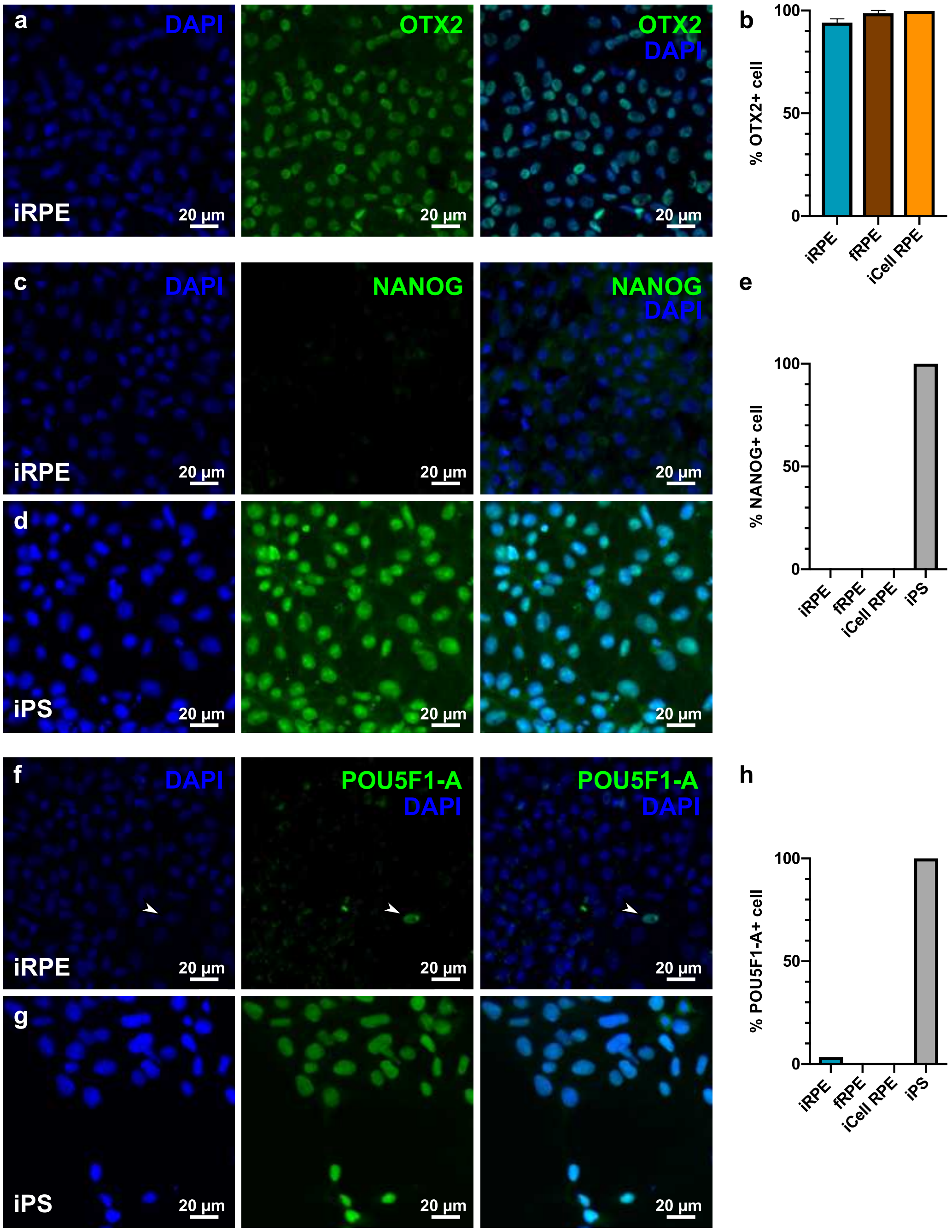

Supplementary Figure 2

Immunolabelling and quantification of OTX2, NANOG and POU5F1-A protein in iRPE. (a) iRPE cells labelled for OTX2 protein. (b) Quantification of OTX2 positive iRPE (n=8), fRPE (n=2) and iCell RPE (n=2) cells from at least 3 images per n. (c) iRPE and (d) iPS cells labelled for NANOG protein. (e) Quantification of NANOG positive iRPE (n=2), fRPE (n=2), iCell RPE (n=2) and iPS (n=2) cells from at least 3 images per n. (f) iRPE and (g) iPS cells labelled for POU5F1-A protein. (h) Quantification of POU5F1-A positive iRPE (n=2), fRPE (n=2), iCell RPE (n=2) and iPS

(n=2) cells from at least 3 images per n. One positive POU5F1-A iRPE cells is signalled by an arrowhead.

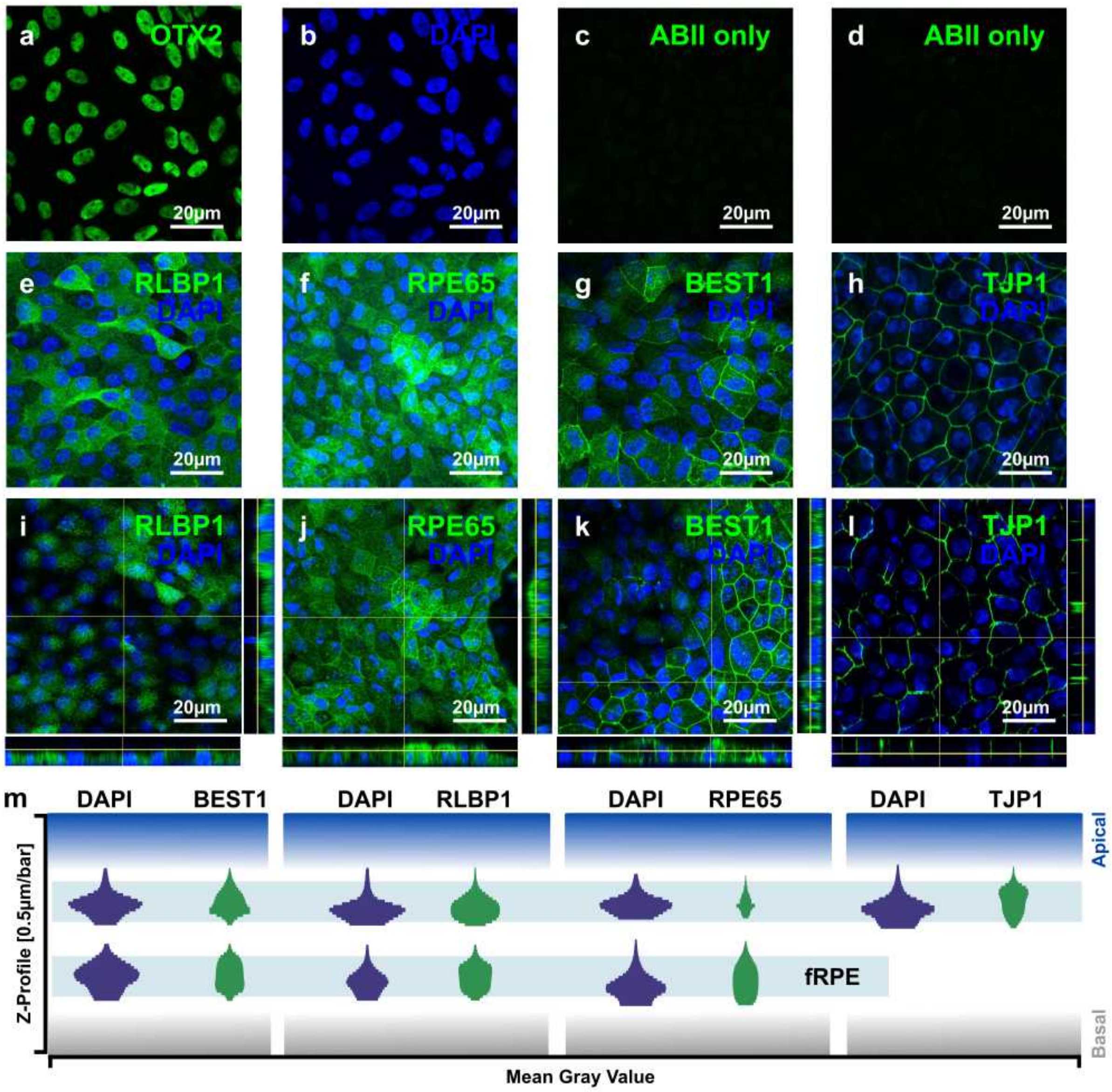

Supplementary Figure 3

Immunolabelling characterization of fRPE. (a-l) fRPE cell line labelled for different RPE markers and imaged with a confocal microscope. Control of background of (b) goat anti-mouse and (c) goat anti-rabbit secondary antibodies incubated alone on fRPE cells. (e-h) Maximum intensity projection of Z-stacks. (i-l) Orthogonal view of Z-stacks for different RPE markers. (m) Violin plots depicting the apical to basal protein distribution.

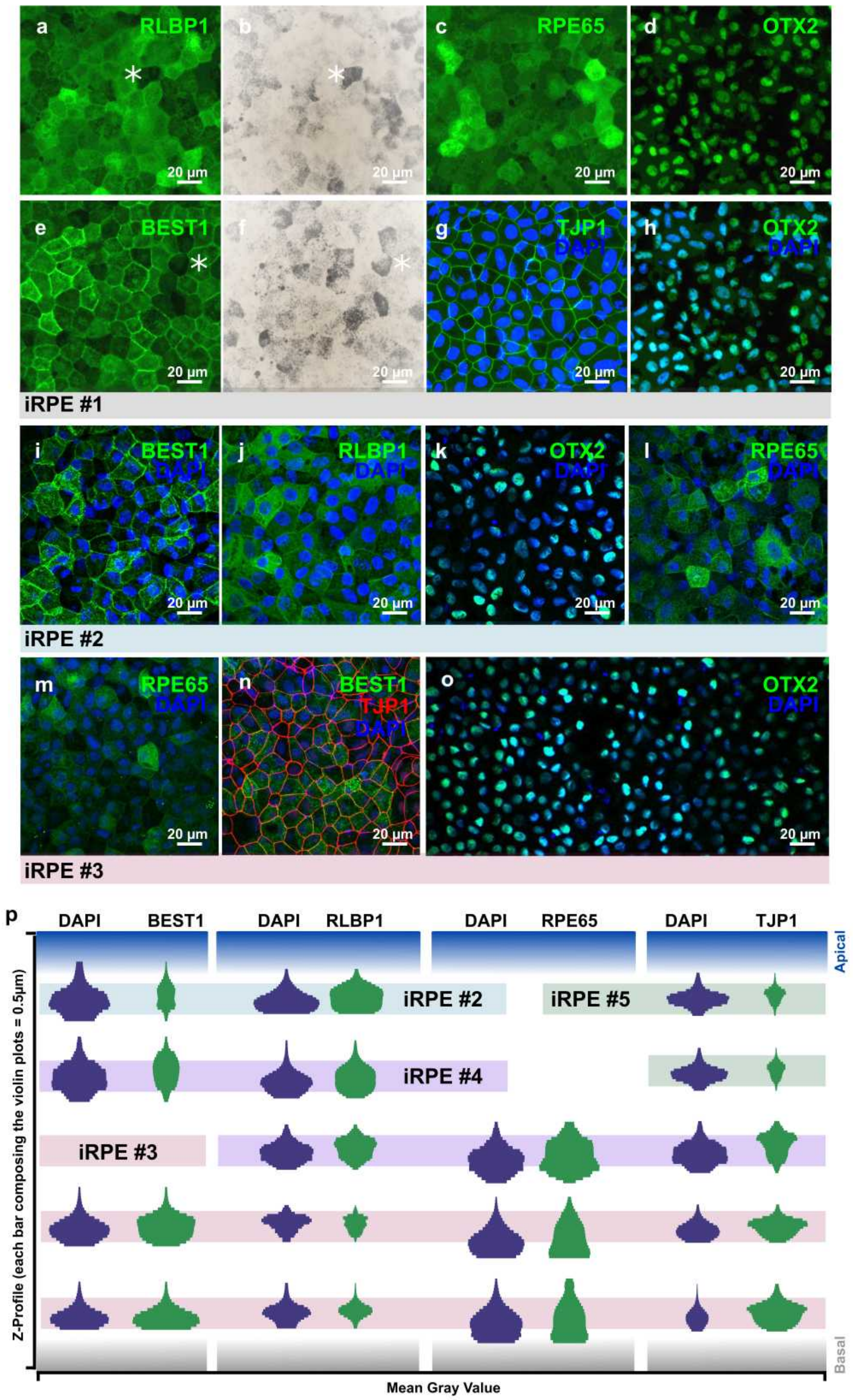

a RLBP1
b
c RPE65
d OTX2
e BEST1
f
g TJP1 DAPI
h OTX2 DAPI
20 µm
iRPE #1
i BEST1 DAPI
j RLBP1 DAPI
k OTX2 DAPI
l RPE65 DAPI
iRPE #2
m RPE65 DAPI
n BEST1 TJP1 DAPI
o OTX2 DAPI
iRPE #3
p
DAPI BEST1 DAPI RLBP1 DAPI RPE65 DAPI TJP1
Apical
iRPE #2
iRPE #5
iRPE #4
iRPE #3
Basal
Z-Profile (each bar composing the violin plots = 0.5µm)
Mean Gray Value

Supplementary Figure 4

Immunolabelling characterization of iRPE. (a to o) iRPE cells from 3 different iRPE lines labelled with antibodies directed against several different RPE markers were analysed with a classical fluorescent microscope (a to h) or a confocal microscope. (a and b, e and f) Note the immunolabelling signal reduction in the most pigmented cells. (i to o) Z-stacks, here displayed as maximal intensity projections. (p) Z-stacks of iRPE cells immunolabelled for RPE markers were acquired with a confocal microscope and the signals from DAPI and the labelled protein were quantified throughout the stacks and displayed as violin plots. iRPE obtained from 5 differentiation protocols started at different times (numbered 1, 2, 3, 4, 5) are shown here to appreciate the protocol reliability.

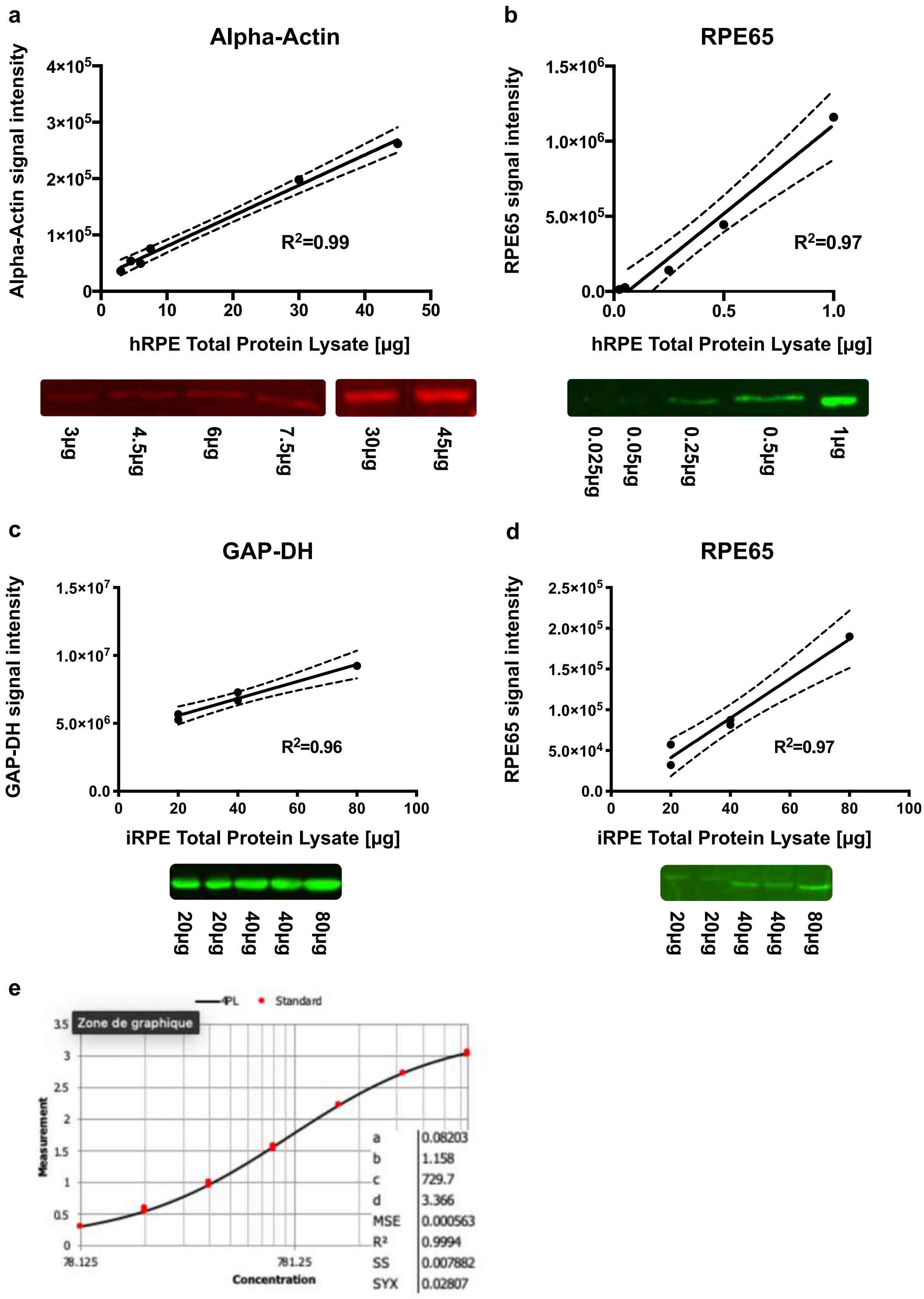

Supplementary Figure 5

Western blot quantification characterization. Standard curve of increasing hRPE (post-mortem adult human sample) total protein lysate

contents showing (a) a linear α-Actin and (b) RPE65 protein signal as determined by linear regression flanked by a 95 % CI. Standard curve of increasing iRPE total protein lysate contents showing (a) a linear GAP-DH and (b) RPE65 protein signal as determined by linear regression. R2: coefficient of determination. p: p-value. (e) Standard curve of the four parameters logistic regression of the RPE65 ELISA (from Figure 7). Y-axis: absorbance at 450nm. X-axis: RPE65 concentration in pg. a, b c, and d: parameters of the 4 parameters logistic curve. MSE: mean squared error. R^2: coefficient of determination. SS: sum of squares. SYX: standard deviation of the residuals.

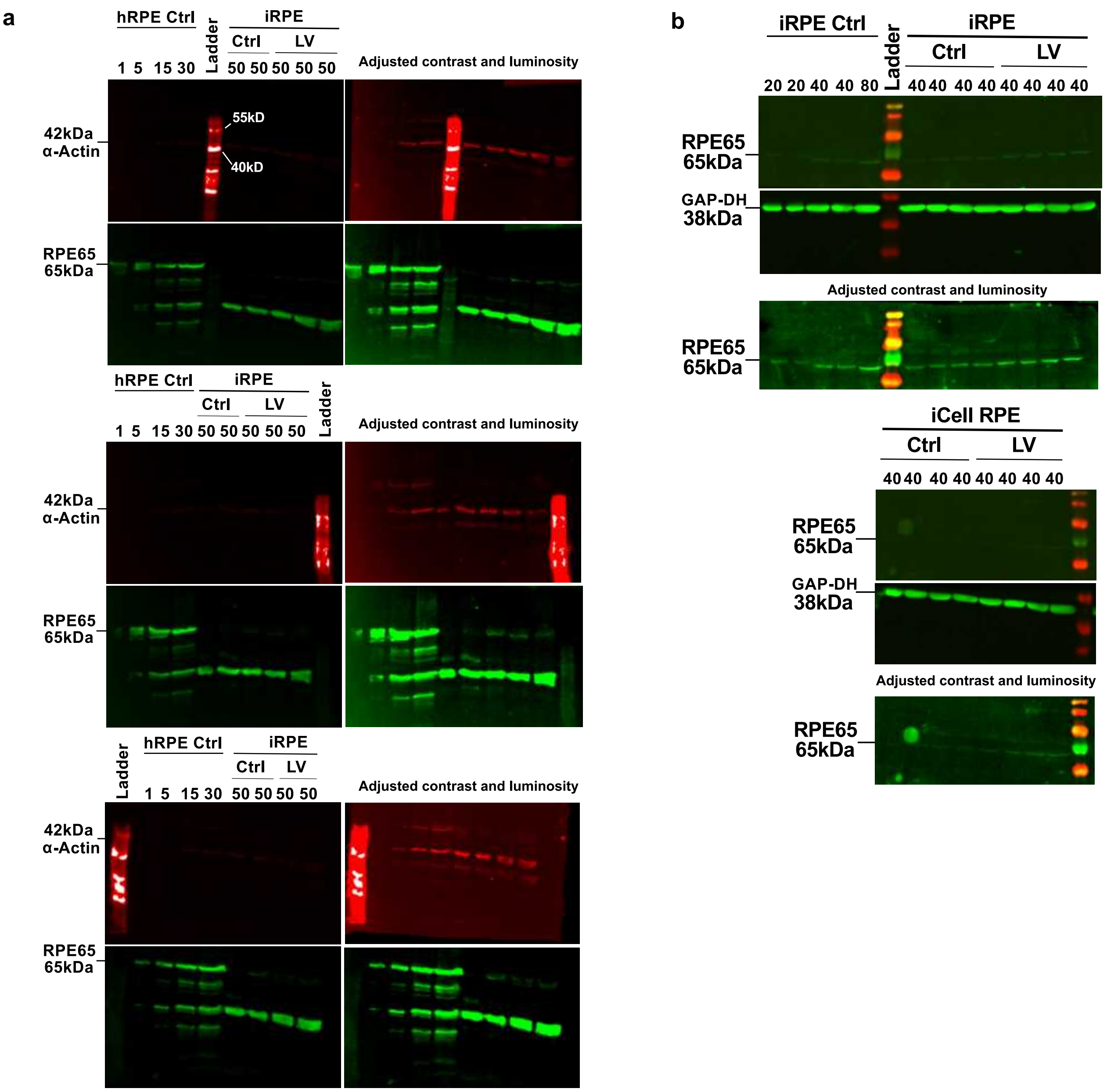

Supplementary Figure 6

Original blots from Figure 6d and 7d. Original blots of the RPE65 gene augmentation experiment described in Figure 6 and 7. (a) Quantification of RPE65 protein (green channel – lower blot) normalized to alpha-actin (red channel – upper blot) was performed by Western blotting and densitometry analysis. Worthy of note, the ladder used is visible in normal light and is partially visible in the red channel, however the size band determination was performed by overlaying the fluorescence channel to the visible one (not shown here). (b) Quantification of RPE65 protein (green channel – upper blot) normalized to alpha-actin (green channel – lower blot) was performed by Western blotting and densitometry analysis. Quantity of loaded protein (in µg) is indicated above the blots.

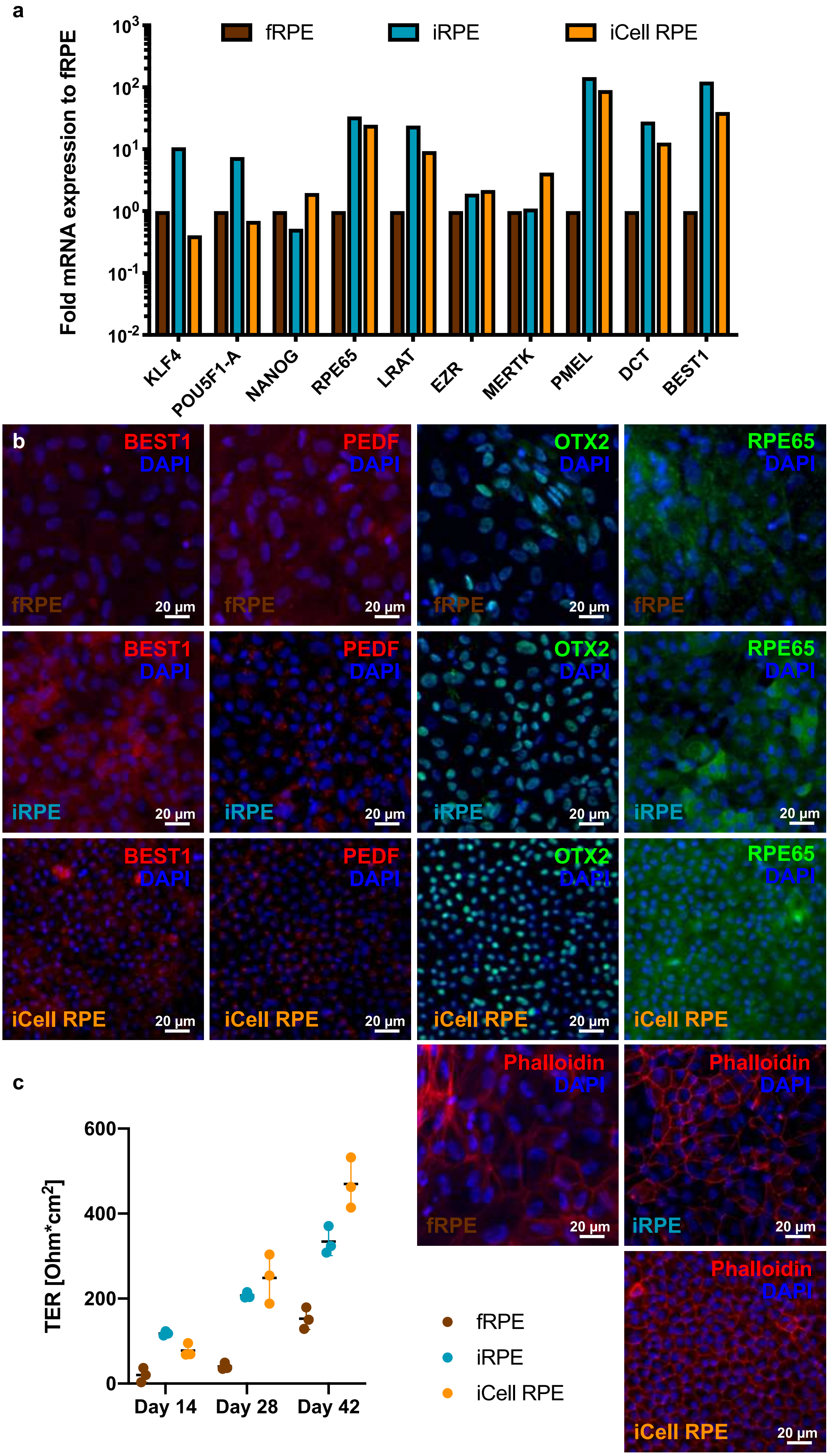
a
Fold mRNA expression to fRPE
fRPE
iRPE
iCell RPE
KLF4
POU5F1-A
NANOG
RPE65
LRAT
EZR
MERTK
PMEL
DCT
BEST1
b
BEST1
DAPI
PEDF
OTX2
RPE65
fRPE
iRPE
iCell RPE
20 µm
c
TER [Ohm*cm2]
600
400
200
0
Day 14
Day 28
Day 42
Phalloidin

Supplementary Figure 7

Expression of RPE markers at mRNA and protein levels. (a) Quantitative PCR investigating RPE marker expression in iRPE (n=1) and iCell RPE (n=1) normalized to fRPE (n=1). (b) iRPE, iCell RPE and fRPE cells immunolabelled for several RPE markers. (c) TER was measured on iRPE, iCell RPE and fRPE monolayers at 14, 28 and 42 days in culture on transwell.

Supplementary Method

1. iRPE differentiation protocol

Day	Manipulations (including final concentrations)
**Pre-protocol**	- iPSCs were cultured in feeder-free conditions in mTeSR medium kit (Ref. 5851 & 5852, StemCell) or E8 medium kit (Ref. A1517001, StemCell) on Embryonic Stem Cell grade Matrigel (Cat.Nb. 354277, Corning).
**Day 0**	- iPSCs (60-80 % confluent) were lifted from the plate using a solution of type IV Collagenase (1 ml/well of 6 well-plate, Ref. 17104-019, LifeTechnologies) at a concentration of 400 U/ml in either DMEM, DMEM-Glutamax or DMEM/F12 (thereafter referred to as "media", for the references see next table, LifeTechnologies).<br>- iPSC colonies were collected into a 15 ml polypropylene tube and broken down into clumps of approximately 50-300 μm using a 5 ml serological pipet by up and down pipetting, from 5 to 15 times avoiding bubbles. The clumps size was checked under microscope every 5 up and down pipetting movements.<br>- iPSC clumps were transferred into T25 culture flasks or P10 culture petri dishes in 10ml Embryoid Body Medium (EBM, see next table for composition) supplemented with the ROCK inhibitor Y-27632 dihydrochloride (5 μM, Ref. CAS129830-38-2/CAT 72304, StemCell).<br>**Important tips**<br>- Two to three wells (of a 6-well plate) of iPSCs were lifted, dissociated into clumps and put in one T25 flask or P10 petri dish.<br>- Lifting with collagenase necessitated an incubation of 20 min to 1 h at 37 °C.<br>- iPSCs were ready to be collected when large iPSC clumps were easily detaching or floating.<br>- iPSC clumps were collected using a 5 ml serological pipet (with blasting of the iPSC clumps still attached to gently detach them. If not detaching, they were not taken).<br>- After collection of iPSCs, the clumps were left to sink in at least 10 ml of any warm medium (approx. 5 min) and the medium was changed before proceeding to clump break down.
**Day 1**	- 500 to 550 EBs were plated on Matrigel Reduced Factor (MRF, dilution 1:30, Ref. 354230, Corning)-coated petri dishes in 5 ml NIM (see next table for composition) for a 6 cm diameter petri dish (density of 20-25 EBs·cm^{-2}).<br>**Important tips**<br>- 6 cm diameter petri dishes were preferred as it facilitates future pigmented foci (PF) dissection.

	- Matrigel-coated plates were incubated at least 30 min at 37 °C or 1 h at room temperature (RT) right before use. If not used immediately, Matrigel-coated plates (before the incubation at 37 °C or RT) were wrapped in Parafilm®, stored at 4 °C and used up to one week later.<br>- EBs were collected using a 5 ml serological pipet and left to sink in a polypropylene tube, the flask or petri dish was washed once with any medium to collect all EBs left.<br>- EBs were left to sink, the medium was discarded and EBs washed once with any medium.<br>- EBs were transferred to a 6 cm diameter petri dish, counted and collected at the same time using a 100-1000 µl micropipette and an under hood microscope (such as the Leica DMS300).<br>- Two or more EBs that merged counted as one EB.<br>- Once 500 to 550 EBs were transferred to the MRF-coated petri dish in NIM, the petri dish was gently shaken in the incubator to distribute the EBs evenly on the plate.
**Days 4 & 7**	- Medium changed to 5 ml fresh NIM.
**Day 10**	- Medium changed to 5 ml fresh Retinal Differentiation Medium with vitamin A (RDMcA, see composition next table).
**Day 13-ON**	- Medium switched to 5 ml fresh Retinal Differentiation Medium without vitamin A (RDMsA, see composition next table)<br>- Medium changed to fresh RDMsA every 2 days.<br>**Important tips**<br>- Pigmentation typically appeared around day 18-24.
**Between Day 30 and 40**	- Once a satisfactory level of pigmentation was reached, PF were manually dissected, collected in a polypropylene tube and plated directly on MRF (1 to 3 PF·cm^{-2}) in RDMsA (2.5-3 ml if 6 cm diameter petri dish).<br>**Important tips**<br>- 6 cm diameter petri dishes were preferred to facilitate future pigmented patches dissection.<br>- The petri dish was extremely well but gently washed before PF dissection and collection.<br>- During dissection, a scalpel was used to cut around the PFs.<br>- Once a PF was cut, a syringe was used to lift it and easily collect it with a 20-200 µl pipette.<br>- PFs were left to sink in a polypropylene tube before changing to fresh RDMsA.<br>- Usually, the 50 best PFs/petri dish were collected and plated in one MRF-coated 6 cm petri dish.
**PF culture**<br>**PP collection (approx. 20 days or whenever a satisfactory expansion of iRPE is obtained)**	- RDMsA (2.5-3 ml) was changed once a week.<br>- Pigmented patches were dissected and collected in a polypropylene tube.<br>- Pigmented patches were left to sink, supernatant was discarded and 0.7 ml Trypsin (Ref. T3924, Sigma-Aldrich) added.<br>- Pigmented patches were incubated 5 min at 37 °C, and gentle pipetting was performed to obtain a single cell dissociation. This step was repeated until satisfactory dissociation was reached (twice usually sufficient, three times may result in cell damage).<br>- Dissociated pigmented patches were centrifuged (115 g, 2-3 min at RT), supernatant was discarded, the pellet resuspended in 1ml RDMsA, and the cell number counted.<br>- iPS-derived RPE (iRPE) cells dissociated from the pigmented patches were plated on MRF-coated (1:30) plate at a density of 0.5 to $1{\cdot}10^5$ cells·cm^{-2} in RDMsA.<br>**Important tips**

	- Non-pigmented tissues neighboring pigmented patches were removed by scraping with a syringe tip, and the petri dish washed extremely well before pigmented patches collection (usually three times with any warm medium).<br>- During dissection, a 20-200 µl pipet was used to scrape and collect pigmented patches.<br>- Trypsin dissociation sometimes resulted in some dying cells and floating DNA that clumped cells together. This clump was sometimes removed with a 1-10 µl pipet.<br>- Dissociated iRPE cells were not filtered to prevent loss of cells.<br>- Cells were considered to be at passage 1 at this stage.
**iRPE subculture**	- Once iRPE cells reached confluence (between days 7 and 15), they were split at a density of 0.5 to $1{\cdot}10^5$ cells$\cdot$cm^{-2} in RDMsA on MRF-coated culture vessel.<br>- If iRPE cells were passaged for expansion, they were split on MRF-coated plate.<br>- If iRPE cells were passaged for assay or characterization, they were split on MRF-coated transwell (12 mm diameter, Cat.Nb. CLS3460, Sigma-Aldrich).<br>- Unless otherwise stated, iRPE cells were matured for 42 days on transwell before assay or characterization.<br>**Important tips**<br>- 12 mm diameter transwells were preferred for trans-epithelial resistance measurements (see TER measurement Supplementary Method) as the medium in the apical compartment of smaller transwells was not covering the electrode properly.

Medium	Compounds (including final concentrations used)
**EBM (Embryoid Body Medium)**	DMEM/F12 (Ref. 11330-032, LifeTechnologies), 1 % N2 supplementation (Ref. 17502-048, LifeTechnologies), 1 % B27 supplementation (Ref. 17504-044, LifeTechnologies), 1 % L-glutamine (Ref. 25030-024, LifeTechnologies), 0.1 mM Beta-Mercaptoethanol (Ref. M7522, Sigma-Aldrich).
**NIM (Neural Induction Medium)**	DMEM/F12 (Ref. 11330-032, LifeTechnologies), 1 % N2 supplementation (Ref. 17502-048, LifeTechnologies), 1 % L-glutamine (Ref. 25030-024, LifeTechnologies), 1 % MEM-NEAA (Ref. 11140-035, LifeTechnologies), 2 µg/ml Heparin (Ref. 375095-100KU, Millipore).<br>**Important tips**<br>- Prefer freshly prepared NIM for every medium change.
**RDMcA (Retinal Differentiation Medium *cum* vitamin A)**	DMEM-Glutamax – F12 Nutrient Ham mix 3:1 proportion (respectively Ref. 32430-027 and 21765-029, LifeTechnologies), 2 % B27 (Ref. 17504-044, LifeTechnologies).
**RDMsA (Retinal Differentiation Medium *sine* vitamin A)**	DMEM-Glutamax – F12 Nutrient Ham mix 3:1 proportion (respectively Ref. 32430-027 and 21765-029, LifeTechnologies), 2% B27 without vitamin A (Ref. 12587-010, LifeTechnologies).<br>**Important tips**<br>- Prefer freshly prepared RDMsA for every medium change.

2. RNA and DNA extraction

2.1 Preliminary information on the protocols used

- For the study of mRNA RPE marker expression (Fig. 2a, Supp.Fig. 1), RNA was extracted with TRI Reagent® (T9424, Sigma-Aldrich) according to manufacturer's protocol and stored at -80 °C until use. Retrotranscribed RNA was stored at -20 °C.
- For the study of the potential leakiness of different promoters in iRPE cells (Fig. 5), gDNA was extracted with the protocol described in section 2.2 and stored at 4 °C until use.
- For the study of RPE65 mRNA expression and lentiviral integration after RPE65 gene augmentation therapy (Fig. 6), RNA and gDNA were extracted from the same iRPE sample with TRI Reagent® (T9424, Sigma-Aldrich) according to manufacturer's protocol. gDNA pellets revealed to be indissoluble and had to be incubated in dH_2O at 37° C for 20 min in a ThermoMixer® C (Eppendorf) at 400 rpm, centrifuged, and the supernatant collected to perform a precipitation protocol detailed in section 2.3. RNA was stored at -80 °C, retrotranscribed RNA at -20 °C. and gDNA at 4 °C.

For all of the above, nucleic acid concentration was measured by a NanoDrop 2000c from ThermoFisher. When 260/280 nm and 260/230 nm absorbance ratio were lower than 1.6 and 1.8 respectively, an additional precipitation step was performed (section 2.3).

2.2 gDNA extraction

This protocol was adapted from[1].
Volumes listed in the following protocol are suitable for 1 well of a 12-well plate; they were changed accordingly to culture vessel surface when needed.

1. Proteinase K (740506, Macherey-Nagel) was added to gDNA extraction buffer to obtain a final concentration of 0.2 mg/ml shortly before use.
2. 500 µl of gDNA extraction buffer was added per well of a 12-well plate (see composition below) and collected in sample tubes.
3. Samples were incubated 2-3 h at 55 °C in a ThermoMixer® C (Eppendorf) at 400-1000 rpm.
4. 250 µl NaCl 5-6 M were added per tube and mixed by inversion (no vortexing from now on).
5. Tubes were centrifuged at maximum speed (at least 12'000 g) for 5 min.
6. Supernatant was discarded, 300 µl isopropanol (Ref. 1.09634.2511, Merck-Millipore) were added and mixed by inverting the tube.
7. Tubes were centrifuged at maximum speed (at least 12'000 g) for 5 min.
8. Supernatant was discarded and the pellets washed twice with 70 % EtOH (Ref. 1.00983.2511, Merck-Millipore).

9. Pellets were left to air dry for 10 min before resuspension in 100 to 500µl dH_2O (according to pellet size).
10. If the pellets were too sticky after resuspension, they were centrifuged at maximum speed (at least 12'000 g) and the supernatant (containing gDNA in solution) transferred to a fresh tube.
11. gDNA samples were stored at 4 °C for a few months (tips: prefer good quality tube to avoid evaporation), or at -20 °C for long-term storage.

gDNA extraction buffer	*Ref*
50 mM Tris-HCl pH 8.0	(Trizma-HCl & -Base T3253, T1503)
100 mM NaCl	(S7653, Sigma-Aldrich)
10 mM EDTA	(1.12029.1000, Merck-Millipore)
NP-40 0.5 %	(BDH, Brunschwig)
0.2 mg/ml Proteinase K	(740506, Macherey-Nagel)

2.3 RNA/DNA precipitation

Volumes listed in the following protocol are suitable for 50 µl of starting material in dH_2O; they were changed accordingly to the initial volume used.

1. dH_2O was added to the DNA or RNA to reach a total volume of 50 µl.
2. 125 µl of EtOH 100%, 5 µl of NaAc (AM9740, Ambion), and 0.1-1 µl of Glycogen (10814-010, Invitrogen) was added per sample.
3. Sample were put at -80 °C for at least 1 h.
4. Sample were centrifuged at maximum speed (at least 12'000 g) at 4 °C for 20 min.
5. Pellets were washed with 500µl 70 % EtOH and left to air dry for 10 min.
6. Pellets were resuspended in dH_2O (volumes depended on starting material).

3. Quantitative polymerase chain reaction

All qPCRs were performed on a LightCycler®96 with the FastStart Essential DNA Green Master mix (Ref. 06402712001) from Roche.

3.1 Programs

For:

- Gene expression analysis (Fig. 2a).
- mRNA expression after RPE65 gene augmentation therapy (Fig. 6).

	Step	Duration	Temperature	T change speed	Back to step	Repeat	Acquisition
1	Preincubation	10min	95°C	4.4°C/sec			
2	Denaturation	10sec	95°C	4.4°C/sec			

3	Annealing	10sec	60°C	2.2°C/sec			
4	Elongation	10sec	72°C	4.4°C/sec	2	40x	Single point
5	Incubation	10sec	95°C	4.4°C/sec			
6	Melting	60sec	65°C	2.2°C/sec			
7	Melting	1sec	97°C	0.5°C/sec			20x/°C
8	Cooling	30sec	37°C	2.2°C/sec			

- LV genome relative copy number quantification after RPE65 gene augmentation therapy (Fig. 6).
- LV genome relative copy number quantification after multiple GFP constructs transduction (Fig. 5).

	Step	Duration	Temperature	T change speed	Back to step	Repeat	Acquisition
1	Preincubation	10min	95°C	4.4°C/sec			
2	Denaturation	10sec	95°C	4.4°C/sec			
3	Annealing	10sec	60°C	2.2°C/sec			
4	Elongation	25sec	72°C	4.4°C/sec	2	40x	Single point
5	Incubation	10sec	95°C	4.4°C/sec			
6	Melting	60sec	65°C	2.2°C/sec			
7	Melting	1sec	97°C	0.5°C/sec			20x/°C
8	Cooling	30sec	37°C	2.2°C/sec			

3.2 Primer list

All primer pairs investigating mRNA expression contained at least one primer designed on an exon-exon junction. All primer pairs used for this study were designed using the Primer3 webtool from NCBI (https://www.ncbi.nlm.nih.gov/tools/primer-blast/) with a targeted optimal melting temperature of 60 °C except for the custom designed β-Actin and LTR primers, and the POU5F1-A primers retrieved from a publication from Wang and colleagues[2].

Gene/Name	Sequence 5'->3'	Amplicon size (in bp)
KLF4 FW	AGAGTTCCCATCTCAAGGCA	106
KLF4 RV	GTCAGTTCATCTGAGCGGG	106
SRP72 FW	CAAAGGAACAAGGACAGGGA	108
SRP72 RV	GCAGCCATCTTTCTGGATCT	108
GPI FW	AAACATGTTCGAGTTCTGGGA	101
GPI RV	GCTCGAAGTTGTCAAAACCC	101
ATP5F1B FW	TGGCCACTGACATGGGTACT	98
ATP5F1B RV	CAAGTCATCAGCAGGCACAT	98
ACTB Locus FW	GTGAACCCTGCAAAAGGGTGG	103
ACTB Locus RV	CCTCTAAGGCTGCTCAATGTCA	103
NANOG FW	CAATGGTGTGACGCAGAAGG	95
NANOG RV	GAAGGTTCCCAGTCGGGTTC	95
POU5F1-A FW	CCCCTGGTGCCGTGAA	97
POU5F1-A RV	GCAAATTGCTCGAGTTCTTTCTG	97
MERTK FW	AAGGCCGCATTGCTAAGAT	121
MERTK RV	CGCGTAGCTATTTCCCACAT	121
LRAT FW	TACTGCAGATATGGCACCCC	106
LRAT RV	CCAAGACTGCTGAAGCAAGA	106
CRALBP FW (RLBP1)	CGAGTGGTCATGCTCTTCAA	110
CRALBP RV (RLBP1)	TCCTCATTCTCCAGCAGCTT	110
RPE65 FW	AAAAATGCCAGAAAGGCTCC	108
RPE65 RV	AGTTGTATTGGGGAGCGTGA	108

MITF-H FW (isoform H)	GATGGAGGCGCTTAGAGTTC	121
MITF-H RV (isoform H)	GCGTGATGTCATACTGGAGG	121
MITF-M FW	CTTTGCCAGTCCATCTTCAA	165
MITF-M RV	ATGTGGTACTTGGTGGGGTT	165
OTX2 FW	CGAGGGTGCAGGTATGGTTT	151
OTX2 RV	TGGCCACTTGTTCCACTCTC	151
BEST1 FW	CCTTGGAAAACAGGGATGAAGC	120
BEST1 RV	AGCTGTATGGCTGTGACTGG	120
PMEL17 FW	GCCTGGCAGTGGTCAGCACC	199
PMEL RV	CGGGGTAGACGCAGCCAGTGA	199
DCT FW (TYRP2)	CCCATTTTTGTGGTTCTTCATTCC	83
DCT RV (TYRP2)	CAGGCATCTGCAGGAGGATT	83
EZRIN FW	ACCGTGGGATGCTCAAAGAT	138
EZRIN RV	GTCCAAGGGCATCAACTCCA	138
LTR 3' FW	CCCTTTCGCTTTCAAGTCCCTGTT	665/323/265
LTR 5' RV	AAGGGCTAATTCACTCCCAACGAAG	665/323/265
ß-Actin FW (ACTB)	TCACCCACACTGTGCCCATCTACGA	295
ß-Actin RV (ACTB)	CAGCGGAACCGCTCATTGCCAATGG	295

3.3 qPCR efficiencies

RPE markers mRNA expressions (Fig. 2a)

Gene	Efficiency calculated (mean ± SD)	N (number of efficiency calculations)	Efficiency used for quantification
SRP72	2.001893 ± 0.10180598	8	2
ATP5B	1.954791 ± 0.10636167	12	1.954791
KLF4	1.944642 ± 0.13141034	4	1.944642
RPE65	1.942735 ± 0.1330765	9	1.942735
LRAT	1.982685 ± 0.16041377	6	1.982685
EZRIN	2.072264 ± 0.1007441	12	2
MERTK	1.992146 ± 0.22606061	9	1.992146
MITF-H	1.900681 ± 0.1017755	9	1.900681
MITF-M	2*	0*	2*
PMEL7	1.930121 ± 0.10921096	12	1.930121
DCT	2.037343 ± 0.21133442	9	2
BEST1	2.013213 ± 0.09277185	9	2
Additional qPCR to assess pluripotency marker expressions			
SRP72	1.728801	1	1.728801
ATP5B	1.759272 ±0.212347691	6	1.759272
POU5F1-A	1.651726 ± 0.13424584	5	1.651726
NANOG	1.667201 ± 0.0189748	4	1.667201

* Not enough positives Cq values to calculate the efficiency from iPS, fRPE or iRPE cells. Efficiency was determined from a 5-fold dilution five-point standard curve using mRNA extracted from an in-house derived melanoma cell line.

LV transduction of multiple GFP constructs (Fig. 5)

Gene	Efficiency	N (number of efficiency calculations)	Efficiency used for quantification
LTR	2	-	2
β–Actin (ACTB)	2	-	2

LV RPE65 augmentation gene therapy (Fig. 6 & 7)

Gene	Efficiency calculated (mean ± SD)	N (number of efficiency calculations)	Efficiency used for quantification
LTR	2.19±1.11	23	2
ACTBL (ACTB)	2.01±0.49	39	2
ATP5B	1.90896533 ± 0.2556605	20	1.90896533
GPI	1.86580864 ± 0.0965892	20	1.86580864
RPE65	1.86414895 ± 0.2129781	16	1.86414895

RPE markers mRNA expressions (Supp. Fig. 7a)

Gene	Efficiency calculated (mean ± SD)	N (number of efficiency calculations)	Efficiency used for quantification
GPI	1.910276 ± 0.12306932	3	1.910276
ATP5B	2.175151 ± 0.07849070	2	2
NANOG	2*	-	2
POU5F1-A	2.084313	1	2
KLF4	2.502993 ± 0.55669293	2	2
RPE65	2.189251 ± 0.24855133	2	2
LRAT	2*	-	2
EZRIN	2.000809 ± 0.002955501	2	2
MERTK	2*	-	2
PMEL7	1.968975 ± 0.03328733	2	1.968975
DCT	1.950708 ± 0.03341305	2	1.950708
BEST1	1.993593 ± 0.010199357	2	1.993593

*by default due to well loss during qPCR

3.4 qPCR experimental design and quantification method

All qPCR quantifications were performed on R free software using a method adapted from Hui & Feng 2013[3]. Statistical analyses and graphs were performed on GraphPad Prism 6 software.

RPE markers mRNA expression (Fig. 2, a; Sup.Fig.1)

- All qPCRs were performed with at least 3 technical replicates either identical or in-dilution.
- Intra-assay primer efficiency was calculated using the mean of the efficiencies determined from the linear regression of the in-dilution technical triplicate.
- For quantification, calculated efficiencies >2 were replaced by 2
- Negative Cq were replaced by 40 (as the programmes are limited to 40 cycles) in the RPE marker mRNA expression study for the following genes: BEST1 (iPS), MITF-H (iPS), MITF-M (iPS, fRPE, iRPE).
- Prior to quantification, qPCR data was screened on the LightCycler®96 SW1.1 software from Roche to confirm amplification specificity through melting peak visual analysis, and PCR products were checked by agarose gel electrophoresis whenever necessary.
- In every qPCR, two types of negative control were performed: 1) without cDNA to check water contamination, and 2) with non-

retrotranscribed RNA to check gDNA contamination. Both only gave negative or aspecific amplification.

LV transduction of multiple GFP constructs (Fig. 5)

- Before sample collection, iRPE and fRPE cells were treated with 10 µg/ml DNase I solution (1-284-932, Roche) 2 h at 37 °C to digest potential plasmid contamination coming from carried over plasmid in lentivirus preparations.
- All qPCRs were performed with at least 3 technical replicates
- Primer efficiency (LTR and β–Actin) was determined using a 5-points and 5-fold dilution between points standard curve.
- Lentiviral integration was determined as the signal from LTR primer pair and relative iRPE or fRPE cells quantity was determined as the signal from the β–Actin (ACTB gene) primer pair.
- Relative template copy quantity at T0 (start of the qPCR – starting quantity) was determined using the following equation:

$$\frac{Q_{Cq}}{E^{Cq}} = Q_{T0}$$

Q_{Cq} = template copy quantity at threshold crossing arbitrarily put to 1
E = efficiency = 2
Cq = number of cycles to threshold crossing
Q_{T0} = template copy quantity at T0 (start of the qPCR)

- Ratio of lentiviral integration to ACTB gene was calculated by dividing LTR Q_{T0} by β–Actin Q_{T0}.
- LTR primer pair is designed to produce amplicons of different sizes according to the amplified target: 665bp for plasmid amplification and 323bp for integrated lentiviral genome (due to the LTR sequence recombination occurring during lentiviral genome integration).
- Prior to quantification, qPCR data was screened on the LightCycler®96 SW1.1 software from Roche to confirm amplification specificity through melting peak visual analysis, and PCR products were checked by agarose gel electrophoresis whenever necessary.

LV RPE65 augmentation gene therapy (Fig. 6)

- Before collection, iRPE cells were treated with 10 µg/ml DNase I solution (1-284-932, Roche) 2 h at 37 °C to digest potential plasmid contamination coming from carried over plasmid in lentivirus preparations.
- Prior to retrotranscription using the High Capacity cDNA Reverse Transcription Kit from Applied Biosystems (Ref. 4368814), RNA samples were treated with DNase RQ1 (M6101, Promega) according to manufacturer's protocol to digest remnant plasmid contamination.
- All qPCRs were performed with at least 3 technical replicates either identical or in-dilution.

- Intra-assay primer efficiency was calculated using the mean of the efficiencies determined from the linear regression of the in-dilution technical triplicate.
- Prior to quantification, qPCR data was screened on the LightCycler®96 SW1.1 software from Roche to confirm amplification specificity through melting peak visual analysis, and PCR products were checked by agarose gel electrophoresis whenever necessary.
- In every qPCR, two types of negative control were performed: 1) without cDNA to check water contamination, and 2) with non-retrotranscribed RNA to check gDNA contamination. Both only gave negative or aspecific amplification.
- LTR primer pair was designed to produce an amplicon of 265bp for integrated lentiviral genome but not for plasmid (due to the LTR sequence recombination occurring during lentiviral genome integration).

4. Immunohistochemistry

For all immunostainings, negative controls without primary antibody but with secondary antibody incubation were performed.

4.1 Immunostaining protocol

1. Cells were fixed with 4 % paraformaldehyde solution (Ref. 441244, Sigma-Aldrich) during 10-20 min at RT in Transwell.
2. Blocking solution was prepared in the meantime: 5 % foetal bovine serum (FBS, decomplemented 45 min at 56 °C, F7524, Sigma-Aldrich), 5 % Normal Goat Serum (NGS, X0907, Dako) and 0.1 % Triton X-100 (T8787, Sigma-Aldrich) in PBS 1X (#1000324, Bichsel).
3. Cells were washed three times with PBS 1X and incubated in blocking solution minimum 2 h at RT.
4. Primary antibody·ies were added in blocking solution (see antibodies list below) and incubated O/N (overnight) at 4 °C.
5. Cells were washed three times for at least 10 min with PBS 1X.
6. Incubation with secondary antibodies was as follows: 1:1000 goat anti-rabbit/mouse AlexaFluor 488/633 (see antibodies list below) in blocking solution 45 min to 2 h at RT in the dark.
7. Cells were washed three times for at least 10 min with PBS 1X in the dark.
8. Cells were stained with DAPI (2-(4-Amidinophenyl)-6-indolecarbamidine dihydrochloride, 10 µg/ml, Ref. 00000010236276001, Sigma-Aldrich) for 1-5 min at RT in the dark and washed once with PBS 1X.
9. The Transwell membrane was cut using a scalpel blade and put on a paper tissue (cells facing upwards) to remove PBS in excess.

10. For each Transwell, a 20 µl drop (for 12 mm ø TW) of Mowiol® 4-88 Reagent (#475904, Calbiochem) was put on a microscope slide and the Transwell membrane was put on the Mowiol drop (cells facing upwards).
11. The Transwell was then covered with a 20 µl drop of Mowiol before putting a coverslip and letting dry O/D (over-day) or O/N.
12. Microscope slides were then stored at 4 °C or at -20 °C for long-term storage.

Antibodies list

Antibody	Company	Cat. Nb.	Dilution	Host
**OTX2**	Abcam	Ab21990	1:500	Rb
**ZO-1**	ThermoFisher	40-2200	1:500	Rb
**RPE65**	From Grimm's lab	Pin5*	1:200	Rb
**CRALBP**	Abcam	Ab15051	1:200	Ms
**BESTROPHIN-1**	Novus-Biologicals	NB300-164	1:200	Ms
**Phalloidin**	LifeTechnologies	R415	1:100	N/A
**NANOG**	CellSignalingTechnology	StemLight kit 9656S	1:500	Rb
**OCT4-A**	CellSignalingTechnology	StemLight kit 9656S	1:500	Rb
**AlexaFluor 488**	ThermoFisher	A-21121	1:1000	Gt
**AlexaFluor 633**	ThermoFisher	A-21072	1:1000	Gt
**AlexaFluor 488**	ThermoFisher	A-11070	1:1000	Gt
**AlexaFluor 633**	ThermoFisher	A-21053	1:1000	Gt

*From Wenzel *et al.*, 2005[4]

4.2 Violin plots of immunostained protein subcellular localization

In order to determine the subcellular localization of investigated proteins, Z-stacks were acquired with a stack thickness of 0.5 µm using a confocal microscope (LSM700, Zeiss). In ImageJ software, the mean grey value of DAPI and stained protein channels were measured for each stack and presented as violin plots.

5. Electron microscopy

hiPSC-derived RPE cells cultured for 45 days on 6.5 mm Transwells were fixed in a 2.5 % glutaraldehyde solution (EMS, Hatfield, PA, US) in Phosphate Buffer (PB 0.1M pH7.4) (Sigma, St Louis, MO, US) during 90 min at RT. Samples were post-fixed by a fresh mixture of 1 % osmium tetroxide (EMS, Hatfield, PA, US) with 1.5 % of potassium ferrocyanide (Sigma, St Louis, MO, US) in PB buffer during 90 min at RT. The samples were then washed three times in distilled water and dehydrated in acetone solutions (Sigma, St Louis, MO, US) of graded concentrations (30 % for 20 min; 70 % for 20 min; 100 % for 1 h; 100 % for 2 h). This was followed by infiltration in Epon (Sigma, St Louis, MO, US) of graded concentrations (33% Epon in acetone for 2 h; 66% Epon in acetone for 2

h, 100% Epon for 4 h; again 100% Epon for 12h) and finally polymerized for 48 h at 60 °C in an oven. Ultrathin sections of 50 nm were cut transversally on a Leica Ultracut (Leica Mikrosysteme GmbH, Vienna, Austria) and collected on a nickel slot grid of 2x1 mm (EMS, Hatfield, PA, US) coated with a polystyrene film (Sigma, St Louis, MO, US). Sections were post-stained with 4 % uranyl acetate (Sigma, St Louis, MO, US) in H_2O during 10 min, rinsed several times with H_2O followed by Coggeshall 0.4 % lead citrate in H_2O (Sigma, St Louis, MO, US) during 10 min and rinsed several times with H_2O.

Micrographs were taken with a FEI CM100 transmission electron microscope (FEI, Eindhoven, The Netherlands) at an acceleration voltage of 80 kV with a TVIPS TemCam-F416 digital camera (TVIPS GmbH, Gauting, Germany). Large montage alignments were performed using the blendmont command-line program from the IMOD software[5].

6. TER Measurements

RPE cells grown on Matrigel Reduced Factor (MRF)-coated Transwells (12 mm diameter, CLS3460, Sigma-Aldrich), as well as control MRF-coated (no cells) Transwells, were fed with fresh RDMsA a few hours before TER measurements (750 µl in apical compartment, 1500 µl in basal compartment). Before measurements, the EVOM2 electrode was bathed in EtOH 75 % for 10 min and pre-warmed at 37 °C in RDMsA for 5 min. Control Transwell resistance, i.e. the MRF-coated Transwell without cells in medium, was measured first, followed by a maximum of 6 iRPE or fRPE Transwells to ensure measurements in less than 3 min after plate removal from the incubator. Each iRPE, iCell RPE or fRPE line was measured in technical triplicate. TER was determined as follows:

iRPE resistance EVOM2 value :	viRPE
Ctrl MRF-only resistance:	MRF ≈ 140-180 Ω
TW surface:	s = 1.12 cm^2
iRPE effective resistance:	eiRPE $\Omega \cdot cm^2$

$$(viRPE - MRF) \cdot s = eiRPE\ \Omega \cdot cm^2$$

7. POS isolation and phagocytosis assay

All solutions and protocols presented in this section are identical to or adapted from Yingyu and Finneman, 2013[6].

7.1 Stock solutions

These solution can be prepared weeks to months before POS isolation.

1. 100 mM glucose: 1.8 g glucose (G5162, Sigma-Aldrich) in dH_2O, filled to 100 ml when solubilised, stored at 4 °C.

2. 200 mM Tris pH 7.2: using Trizma system from Sigma-Aldrich, 2.808 g Trizma-HCl (T3253, Sigma-Aldrich) + 0.268 g Trizma-Base (T1503, Sigma-Aldrich) in dH_2O, filled to 100 ml when solubilised, stored at RT.
3. 50 mM Taurine: 0.625735 g of Taurine (T8691, Sigma-Aldrich) in dH_2O, filled to 100 ml when solubilised, stored at -20 °C.
4. 20 mM $MgCl_2$: 0.19042 g of MgCl2 (M4880, Sigma-Aldrich) in dH_2O, filled to 100 ml when solubilised, store at RT.
5. 75 % sucrose: 75 g sucrose (1.07687.1000, Merck-Millipore) gradually added in dH_2O 40 ml on stirring/heating plate, filled to 100 ml when solubilised, stored at 4 °C.

7.2 Working solutions

These solution preparations presented here are intended for POS isolation of 16 bovine eyes but the volumes actually prepared were changed accordingly when necessary.

Ideally a linear sucrose gradient should have been made, however lacking the proper material, the following method revealed to be sufficient to obtain satisfying results.

A nine-increment sucrose gradient was prepared as follows (in 2 ultracentrifugation OptiSeal® 30 ml tubes, ref. 361625, Beckman Coulter):

a. Solution a was prepared: 50 % sucrose solution: 16 ml 75 % sucrose + 2.4 ml glucose 100 mM + 2.4 ml Tris pH 7.2 200 mM + 2.4 ml Taurine 50 mM + 0.8 ml dH_2O.
b. As well as solution b: 25 % sucrose solution: 8 ml 75 % sucrose + 2.4 ml glucose 100 mM + 2.4 ml Tris pH 7.2 200 mM + 2.4 ml Taurine 50 mM + 8.8 ml dH_2O.
c. The following mixes were performed: 4.2 ml (a) + 0.6 ml (b), 3.6 ml (a) + 1.2 ml (b), etc…
d. This resulted in 9 mixes of decreasing sucrose percentage: 50 %, 46.875 %, 43.75 %, 40.625 %, 37.5 %, 34.375 %, 31.25 %, 28.125 % and 25 %.
e. 2.4 ml of 50 % sucrose solution were added to each ultracentrifugation tube in a holder to keep them straight and put at -20 °C for 30 min.
f. 2.4 ml of 46.875 % sucrose solution were added to each ultracentrifugation tube in a holder to keep them straight and put at -20 °C for 30 min.
g. Continued likewise until 25 % sucrose solution was added and the tubes were left in the -20 °C freezer
h. The sucrose gradient tubes were put at 4 °C the evening before POS isolation.

Homogenization solution (for 16 eyes, 60 ml, final 20 % sucrose):

a. 16 ml of 75 % sucrose solution + 6 ml Tris pH 7.2 200 mM, + 6 ml Taurine 50 mM + 6 ml $MgCl_2$ 20 mM + 6 ml glucose 100 mM + 20 ml dH_2O, stored at 4 °C.

WASH solutions (3 wash solutions, 50, 25 and 10 ml)

1. 5 ml Tris pH 7.2 200 mM + 5 ml Taurine 50 mM + 40 ml dH_2O, stored at 4 °C.
2. 2.5 ml Tris pH 7.2 200 mM + 2.5 ml Taurine 50 mM + 3.3 ml of 75 % sucrose solution + 16.7 ml dH_2O, stored at 4 °C.
3. 1 ml Tris pH 7.2 200 mM + 1 ml Taurine 50 mM + 8 ml PBS, stored at 4 °C.

Resuspension solution

b. 0.5 ml sucrose 75 % in 14.5 ml any high glucose (4.5 g/l) DMEM (final concentration: 2.5 % sucrose), stored at 4 °C.

7.3 POS isolation process

All solutions needed for the isolation process were prepared at least the day before.

1. 16 bovine eyes were obtained from the closest slaughterhouse (Bell slaughterhouse, Cheseaux-sur-Lausanne, Switzerland).
2. Eyes were kept on ice and in the dark and were processed maximum 6 h post-mortem.
3. 10 ml of homogenization solution in a 50 ml tube were chilled on ice, as well as the rest of the homogenization solution and an empty conical tube.
4. The following tools were prepared: big dissection scissors, a scalpel with a small pointy blade, a small plier with flat extremity to peel the retina off, some gauze, two big polystyrene boxes with a cover and ice inside (one for the eyes, one for the homogenization solution and conical tubes to collect the retina) and a basin with a plastic bag to collect dissected eyes.
5. Dissection was done under dim red light in order to avoid bleaching of rhodopsin.
6. Before starting dissection, the ultracentrifuge (Optima L-90K, Beckman Coulter) was set to run with the empty rotor (70Ti, Beckman Coulter) at 3000 rpm to reach 4 °C.
7. Each eye was dissected as follows: the eye was held by the extra-ocular muscles and its side was pierced with a scalpel at the cornea-sclera interface (photos 1-3).
8. The anterior segment was cut circularly and the lens discarded, resulting in a half eye cup (photos 3-4).
9. Two small incisions on the opposite side of the eye cup were performed (photo 5) in order to turn the eye cup inside-out like a sock (photo 6).

10. The retina was carefully peeled off the RPE and tapetum with the plier, from the outside towards the optic nerve head until it hung only held by the optic nerve (photo 7).
11. The optic nerve was cut with the scissors to make the retina fall into the pre-chilled 10 ml homogenization tube (photo 8).
12. Once all the retinas were collected, the tube was gently shaken.
13. The retina homogenate was passed through 1 layer of gauze 3 times to remove large fragments (end of procedure in dim red light).
14. An equal volume of retina homogenate was laid on top of the sucrose gradients.
15. The gradients were balanced with the homogenization solutions and centrifuged at 112.398 g for 48 min at 4 °C (33’100 rpm with the 70Ti rotor).
16. The pinkish layer in the middle of the tube was collected using a long needle and a 5 ml syringe.
17. The collected layers containing the POS were divided in 4 tubes adapted for the SS-34 rotor (Sorvall, ThermoFisher) and diluted with 5 volumes of WASH 1.
18. The tubes were centrifuged at 3000 g for 10 min at 4 °C (5000 rpm with the SS-34 rotor, in a Sorvall RC6+ from ThermoFisher).
19. Supernatants were discarded; the pellets were resuspended in 5 ml WASH2 and combined to end up with 2 tubes.
20. The tubes were centrifuged at 3000 g for 10 min at 4 °C (5000 rpm with the SS-34 rotor).
21. Supernatants were discarded; the pellets were resuspended in 5 ml WASH3 and combined to end up with 1 tube.
22. The tube was centrifuged at 3000 g for 10 min at 4 °C (5000 rpm with the SS-34 rotor).
23. Supernatant was discarded; the pellet was resuspended in 1ml DMEM 2.5 % sucrose.
24. 1/1000 and 1/10’000 dilutions were made to count POS number using a Neubauer chamber.
25. Step 24 was repeated three times independently.
26. Aliquots of POS of $5 \cdot 10^6$ or $1 \cdot 10^7$ POS were prepared and stored at -80 °C.

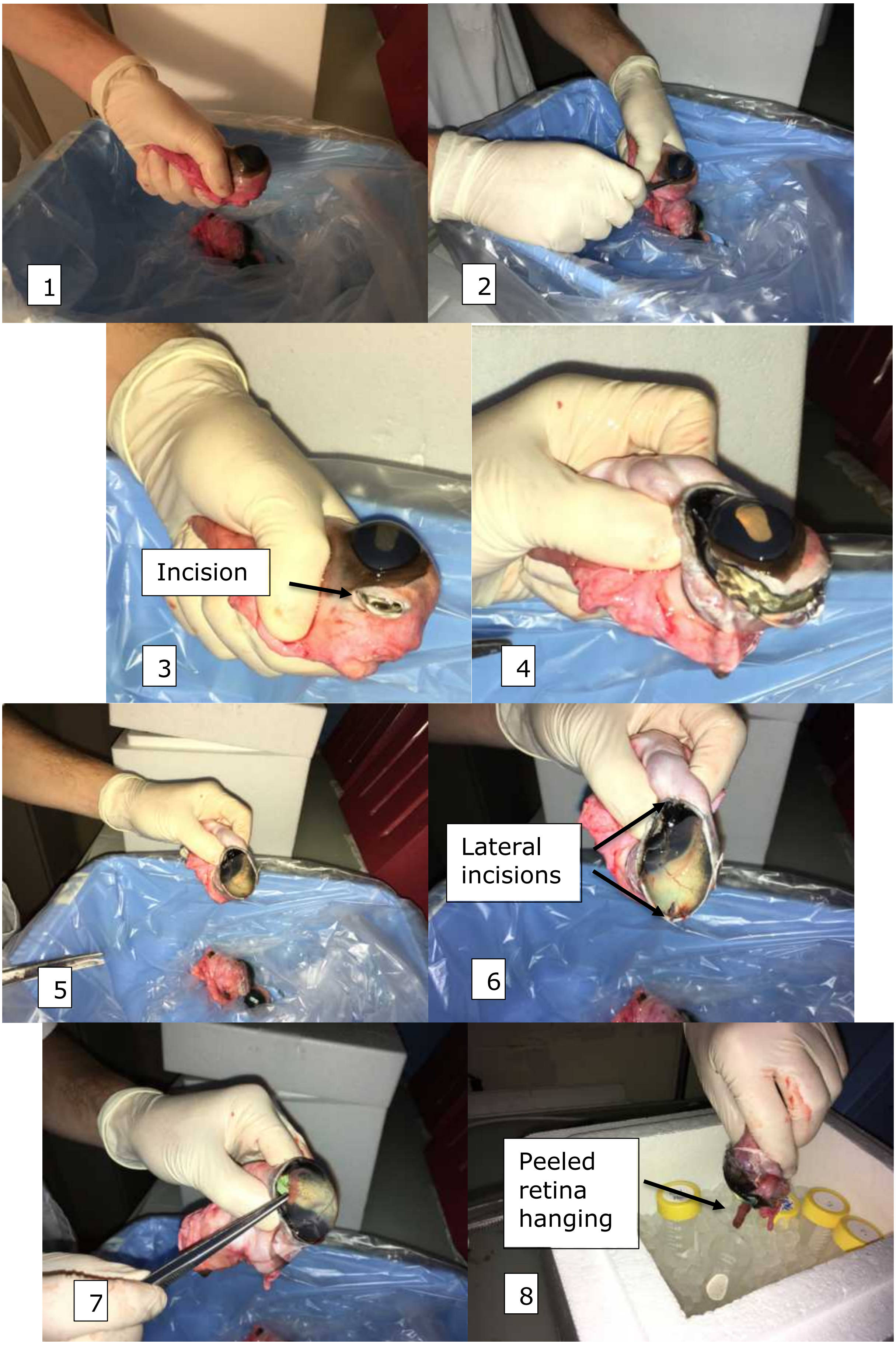
1
2
Incision
3
4
5
Lateral incisions
6
7
Peeled retina hanging
8

7.4 Phagocytosis assay

1. 10 mg of FITC-Dextran (FD2000S, Sigma-Aldrich) were solubilised in 2 ml of $NaHCO_3$ (S5761, Sigma-Aldrich) buffer 0.1 M at pH 9.5 (in a 50 ml conical tube because FITC powder has a tendency to stick to plastic so very difficult in a small tube).
2. The solution was vortexed until no more clumps were present and filtered with 0.2 µm pore size filter (28145-501, VWR Internationals).
3. $5 \cdot 10^6$ POS were taken out of the -80°C for each Transwell to test and thawed in hand.
4. POS were centrifuged 5 min at 3000 g at RT.
5. Supernatant was discarded; the FITC solution was added to POS and mixed by pipetting up and down.
6. POS were put to rotate in the dark for 1-2 h at RT at 20 rotations/min on a tube revolver (Ref. 88881002, ThermoFisher).
7. POS were centrifuged at 3000 g 5 min, the supernatant discarded, and 20 % sucrose in PBS with 10 mM Taurine (solution filtered previously) was used to resuspend the POS.
8. Step 7 was repeated.
9. The POS were resuspended in RDMsA (500 µl RDMsA/Transwell, see RPE differentiation protocol SM for details).
10. 500 µl of the POS solution were added per iRPE or fRPE Transwell and 500 µl of RDMsA was added to control iRPE or fRPE Transwell receiving no POS.
11. The cells were incubated 16h.
12. 3 gentle washes with PBS-CM (PBS #1000324, Bichsel supplemented with 1 mM $MgCl_2$, M4880, Sigma-Aldrich and 0.2 mM $CaCl_2$, C7902, Sigma-Aldrich) were made.
13. Cells were fixed in 4 % paraformaldehyde during 20 min at RT in the dark.
14. Cells were washed with PBS-CM twice.
15. Cells were quenched with 50 mM NH_4Cl (09711, Fluka, made fresh) in PBS-CM for 20 min at RT in the dark.
16. Cells were washed with PBS-CM twice.
17. Transwells were cut and mounted on microscope slides in Mowiol 4-88 Reagent (#475904, Calbiochem).

7.5 Rhodopsin staining

In order to distinguish between microvilli-bound and internalized POS, a Rhodopsin staining was performed resulting in the internalized POS only FITC-positive, and the microvilli-bound POS positive for FITC and Rhodopsin labelling with the secondary Alexa 633 antibody

1. After step 16, the Transwell membranes were cut into two parts with dissection scissors.

2. Both Transwell parts were blocked in PBS-CM with 5 % FBS (F7524, Sigma-Aldrich) and 5 % NGS (X0907, Dako) for at least 1 h in the dark at RT.
3. The Transwell parts were incubated for 40 min in the dark with Rhodopsin antibody (MAB5356, Chemicon) at 1:250 dilution added in blocking solution.
4. Three washes with PBS-CM were performed for 5 min each.
5. Secondary antibody Alexa Fluor 633 Goat-anti-Mouse (A-21053, ThermoFisher) was added to both TW parts at a 1:1000 dilution, 30 min in blocking at RT in the dark.
6. Two washes with PBS-CM were performed for 5 min each.
7. Nuclei were stained with a DAPI solution (10 µg/ml) (000000010236276001, Sigma-Aldrich) 1 min.
8. One wash with PBS-CM was performed for 5 min.
9. Transwell parts were mounted on a microscope slide in Mowiol 4-88 Reagent (#475904, Calbiochem).

8. Lentiviral Production and Titration

8.1 Production

Day 1
HEK293T cells (passage ranging from 3 to 20) were seeded in 10 cm^2 petri culture dishes at a seeding density of $4 \cdot 10^5$ $cells \cdot cm^{-2}$ in HEK media (DMEM-Glutamax with 10 % FBS and 1 % Penicillin-Streptomycin).

Day 2
Transfection solutions were prepared as follows in Falcon® polystyrene conical tube:

	Per culture dish
Mix 1	500 µl HBS 2X
Mix 2	125 µl $CaCl_2$ 1 M<br>x µl Plasmid Mix<br>375-x µl dH_2O cell culture grade

Mix 2 was added to mix 1 drop by drop under continuous mixing by injecting air bubbles in mix 1 and incubated 5 min at RT. The mixed transfection solution was then added drop by drop to the culture dishes (1ml/culture dish). Medium was changed 6-8 h later.

Plasmid mix comprises 10 µg of packaging plasmid, 5 µg of envelope plasmid and 10 µg of one of the following transgene constructs per culture dish.

Plasmid	Type	References
ΔR8.74	Packaging	Naldini *et al*., 1996[7]
pMD2.G	VSV-G Pseudotyped envelope	Naldini *et al*., 1996[7]
pCCL-R0.8-RPE65-	Transgene construct	Matet *et al*., 2017[8]

WPRE4 (Fig.6-7)		
hLox-Ro.8-GFP-WPRE (Fig.5)	Transgene construct	Bemelmans *et al.*, 2006[9]
hLox-GFAP-GFP-WPRE (Fig.5)	Transgene construct	Unpublished – The human GFAP promoter (-2151 to + 48, +1 being the start site of nucleotide accession number M67446) was amplified by PCR, and subcloned in pGEM®-T vector (A3600, Promega) following supplier instructions. The promoter was then digested using ClaI and MluI restriction enzymes and subcloned in Hlox-EFS-GFP-WPRE backbone in place of the EFS promoter.
hLox-EFS-GFP-WPRE (Fig.5)	Transgene construct	Kostic *et al.*, 2003[10]
hLox-Rho-GFP-WPRE (Fig.5)	Transgene construct	Kostic *et al.*, 2003[10]
SIN-ratNSE-AcGFPnuc-WPRE (Fig.5)	Transgene construct	Delzor *et al.*, 2012[11]
SIN-Synapsin-GFP-WPRE (Fig.5)	Transgene construct	Unpublished - The human synapsin promoter (-422 to +53) was cloned in a pENTR/D-TOPO plasmid (Invitrogen) and an LR clonase was performed with the SIN-Gateway-GFP-WPRE plasmid to generate the SIN-synapsin-GFP-WPRE transfer vector).

Day 4

Culture supernatants were collected, filtered with Stericups (0.22 um) and centrifuged twice (70’000 g, 90 min, 4 °C) to pellet then wash the lentivirus that was finally resuspended in PBS with 0.75 mg/ml BSA, aliquoted and stored at -80 °C until use. The virus solution was thus concentrated around 1’000 times.

**Product**	**Provider**	**Ref. Number**
DMEM-Glutamax	LifeTechnologies	32430-027
Foetal Bovine Serum (FBS)	Sigma-Aldrich	F7524
Penicillin/Streptomycin (P/S)	Sigma-Aldrich	P4458
Polystyrene conical tube	Falcon	352073
CaCl2	Sigma-Aldrich	C7902
MgCl2	Sigma-Aldrich	M4880
HEPES Buffered Saline 2X (HBS)	Sigma-Aldrich	51558
PBS	Sigma-Aldrich	D8662
Millipore® Stericup PVDF Filter 0.22µm	Sigma-Aldrich	Z660523
Trypsin	Sigma-Aldrich	T3924
Paraformaldehyde	Sigma-Aldrich	441244
Bovine Serum Albumin Fraction V (BSA)	Applichem	A1391
DNase I	Roche	1-284-932
**Device**	**Company**	**Ref. Number**
Centrifuge	Beckman Coulter	Optima L-90K

Rotor	Beckman Coulter	SW32-Ti
Centrifugation tubes	Beckman Coulter	358126

8.2 Titration

Lentivirus titration was performed by fluorescence-activated cell sorting (FACS) for lentivirus bearing constructs expressing a fluorescent protein, and/or qPCR as well as ELISA against p24 capsid protein (Ref. 0801111, Zeptometrix) according to manufacturer's protocol.

8.2.1 FACS or qPCR titration

Day 1

HEK293T cells were seeded in 12-wells culture plate at a seeding density of 80'000 cells/well at the end of the day.

Day 2

Seeded HEK293T were transduced with different lentivirus doses (at least 3 different quantities, usually ranging from 0.001 to 1 µl) in duplicate in the morning.

Day 5 - FACS

HEK293T cells were dissociated with trypsin and collected in HEK medium (see recipe in Production section), pelleted by centrifugation and resuspended in one volume of PBS, to which one volume of 4 % paraformaldehyde was added. After 10-20 min fixation at RT, HEK293T cells were centrifuged, and resuspended in PBS, filtered in Falcon® FACS tube with 35µm cell strainer cap ready for cell sorting. FACS analyses were performed on a FACSCalibur Cell Analyzer (BD Biosciences).

The titre is determined as follows (TU=Transducing Unit):

$$Titre\ [TU \cdot ml^{-1}] = \frac{\%\ GFP^{+} cells \cdot 80'000}{LV\ quantity\ [ml]}$$

Day 4/5 (at confluence) - qPCR

Each well of HEK293T cells was split to one well of a 12-wells culture dish at a 1:4 ratio in HEK medium supplemented with 1 mM of $MgCl_2$ and 10 µg/ml of DNase I to digest potential plasmid contamination carried over from lentiviral preparation.

Day 7/8 (at confluence) – qPCR

HEK293T cells were collected and gDNA extracted as detailed in section 2.2. Quantitative PCR was run in technical duplicates to quantify the proportion of integrated lentiviral genome (LTR primers – details in section 3.2) to HEK293T genome (β-Actin or ACTBL primers – details in section 3.2). Standard samples were run alongside to produce a standard curve as a reference to determine the titre of the lentivirus production tested.

The standard sample consisted of HEK293T cells transduced with a 3-point 10-fold serial dilution between points of hLox-EFS-GFP-WPRE lentivirus and titrated by FACS as described here. The copy number of integrated lentiviral genome in standard samples was inferred from the proportion of GFP-positive cells using the Poisson distribution. LTR Cqs were subtracted from ACTB Cqs and a standard curve as well as its equation was calculated using Excel exponential trend curve tool.

9. Western Blot

RPE65 protein level was determined by western blotting (Fig. 6d, 6e, 7c, 7c). Post-mortem human RPE total protein lysate was used as a positive control and to produce the standard curves for the verification of signal linearity for RPE65, α-Actin and GAPDH protein. Quantification of RPE65, α-Actin and GAPDH protein content in RPE samples was calculated from the equation of the linear regressions of hRPE or iRPE standard curves (Supp. Fig. 5a-d).

9.1 Protein extraction and quantification

iRPE, fRPE and iCell RPE cells

RPE cells (P3, 42 days in culture on 12 mm diameter Transwells at time of lentiviral transduction, 47 days at collection for figure 6 or 28 days at transduction and 42 days at collection for figure 7) were collected by scraping in 50µl ice-cold lysis buffer (see detailed composition below), vortexed every 3 min during 20 min on ice, centrifuged 30 min at 16.1g. The supernatant (total protein lysate) was transferred into a fresh tube.

hRPE cells

Tissue from human eye globes were obtained following procedures conformed to the tenets of the Declaration of Helsinki for biomedical research involving human subjects and according to the ethical approval (protocol N°340-15) and Swiss law. A post-mortem (< 16 h) eye globe was dissected and human choroidal-RPE samples were stored at -80°C until use. Choroidal-RPE was homogenized in a 1 µl : 1 mg volume : weights ratio (e.g. 100 µl for 100 mg) of ice-cold lysis buffer with a sterile plastic pestle, passed through a 20 G syringe several times, and vortexed on ice. After centrifugation at 16.1 g, the supernatant (total protein lysate) was transferred to a fresh tube.

For both iRPE and hRPE cells, protein concentration in total protein lysate was determined by BCA assay using the ThermoFisher kit (Ref. 23235) following manufacturer's protocol. Sample buffer is added to all samples at a (sample buffer) 1:4 (protein lysate) ratio, incubated at 95 °C for 10 min and stored at -20 °C.

Lysis buffer

150 mM NaCl	S7653, Sigma-Aldrich

50 mM Tris pH 7.8	Trizma-HCl & -Base T3253, T1503
1 mM EDTA	1.12029.1000, Merck
1 % Triton X-100	T8787, Sigma-Aldrich
0.04 % SDS	L3771, Sigma-Aldrich

The lysis buffer was freshly supplemented with protease and phosphatase inhibitor cocktail (P8340 and P0044 respectively, Sigma-Aldrich) at a 1:100 ratio before sample protein extraction.

<u>Sample buffer</u>

**Final Concentration**	**For 20 ml**	**Stock Solution**	**Ref**
62.5 mM Tris-HCl pH 6.8	4.1 ml Tris pH 6.8 1.5 M	310 mM	Trizma-HCl & -Base T3253, T1503, Sigma-Aldrich
2 % w/v SDS	2 g	10 %	L3771, Sigma-Aldrich
10 % w/v Glycerol	11.8 ml Glycerol 85%	50 %	104094, Merck
0.01 % w/v Bromophenol blue	1ml Brom. Blue 1%	0.05 %	1610404, BioRad
100 mM DTT	1.54 g	500 mM	A2948.0005, Applichem
Water	3.1 ml		

9.2 Gel electrophoresis and protein transfer

Forty to fifty µg of iRPE total protein lysates were run on a 10 % acrylamide gel in 1X Transfer buffer (see details below) and transferred to a PVDF membrane (IPFL-00010, Immobilon, Millipore) using a BioRad Trans-blot Turbo Transfer System (25 V, 30 min). Prior to the transfer, the PDVF membrane was soaked in absolute methanol for 5 min and the transfer sandwich (gel + PVDF membrane) was done with Whatman® chromatography paper (3030-392, GE) soaked in 1X Transfer buffer.

<u>Gels and buffers composition</u>

**Component**	**Resolving Buffer**	**Stacking Buffer**	**Ref**
Tris Base	182 g	61 g	Trizma-Base, T1503, Sigma-Aldrich
SDS 0.4%	4 g or 20 ml (of a 20 % solution)	4 g or 20 ml (of a 20 % solution)	L3771, Sigma-Aldrich
Water	1000 ml	1000 ml	

Component	Resolving Buffer	Stacking Buffer	Ref
Concentrated HCl	Adjusted to pH 8.8	Adjusted to pH 6.8	1.00316.1000, Merck-Millipore

Component	10 % Resolving gel	4 % Stacking gel	Ref
dH_2O	4.8 ml	6.4 ml	
Resolving buffer	2.5 ml	-	
Stacking buffer	-	2.5ml	
Acryl:Bisacryl 29:1 (40 %)	2.5 ml	1 ml	A7802, Sigma-Aldrich
Ammonium persulfate solution 10 %	100 ul	75 ul	1.01201.0100, Merck
TEMED	5 ul	10 ul	A1148.0025, Applichem

Running and transfer buffers

Component	Running 10x	Transfer 10x	Ref
Tris Base	60.6g	30.3g	Trizma-Base, T1503, Sigma-Aldrich
Glycine	288g	144g	G8898, Sigma-Aldrich
Water	2000ml	1000ml	
SDS	20g	-	L3771, Sigma-Aldrich

One volume of 10X Running buffer was diluted in 9 volumes of dH_2O to obtain 1X Running buffer. One volume of 10X Transfer buffer was diluted in 7 volumes of dH_2O and 2 volumes of absolute methanol (106009, Merck-Millipore) to obtain 1X Transfer buffer.

9.3 Blot staining and imaging

The membrane was blocked in Odyssey® TBS Blocking Buffer (P/N 927-50100, Licor) for 2-4 h at RT, incubated O/N at RT with RPE65 (NB100-355, Novus Biologicals) and α-Actin (A2066, Sigma-Aldrich) primary antibodies in blocking buffer with 0.1 % Tween-20 (937773, Fluka), washed three times in TBS containing 0.1 % Tween-20 (TBST) for 5 min, incubated with near infra-red (NIR) secondary antibodies (goat anti-rabbit 800nm: 926-32211, goat anti-mouse: 926-68070, both from Licor) and washed again three times in TBST for 5 min. Blot imaging was performed

with an Azure c600 imaging system (5 min exposition for both NIR channels).

9.4 RPE65 protein quantification

A serial dilution of human primary RPE total protein lysate was used to produce a standard curve and verify RPE65 and α-Actin signal linearity. Band intensities were measured with the Image Studio Lite software from Licor (version 5.2.5). Background parameters were set as follows (Median, Top/Bottom, 3 pixels width). Determination coefficients of the linear regressions of the standard curves (Sup.Fig. 4a and b) confirmed signal linearity. RPE65 and α-Actin signal quantifications were determined from the equation of the linear regressions of the control standard curves and RPE65 protein signal was then normalized to α-Actin.

10. ELISA

Total protein lysate of iRPE, iCell RPE of fRPE cells was obtained as described in section 9 (Western Blot). ELISA was performed according to the manufacturer's protocol (ref. XPEH2516 from XpressBio, USA). The total protein lysate of each sample was diluted in so-called sample dilution buffer from the kit 1/100 before use. The RPE65 standard from the kit was diluted in sample dilution buffer containing 1/100 of lysis buffer to assess any effect of the lysis buffer. Absorbance at 450nm to quantify RPE65 was performed with an Envision 2105 Multimode Plate Reader. Standard curve regression was performed with the MyAssay webtool using a 4-parameter logistic (4PL) regression curve (Supp. Fig. 5e) and the RPE65 concentration of the samples was determined from the equation of the curve.

References

1. Lain, E. *et al.* A novel role for embigin to promote sprouting of motor nerve terminals at the neuromuscular junction. *J. Biol. Chem.* **284**, 8930–8939 (2009).
2. Wang, X. *et al.* Alternative translation of OCT4 by an internal ribosome entry site and its novel function in stress response. *Stem Cells* **27**, 1265–1275 (2009).
3. Hui, K. & Feng, Z. P. Efficient experimental design and analysis of real-time PCR assays. *Channels* **7**, 160–170 (2013).
4. Wenzel, A., Grimm, C., Samardzija, M. & Remé, C. E. Molecular mechanisms of light-induced photoreceptor apoptosis and neuroprotection for retinal degeneration. *Prog. Retin. Eye Res.* **24**, 275–306 (2005).
5. Kremer, J. R., Mastronarde, D. N. & McIntosh, J. R. Computer visualization of three-dimensional image data using IMOD. *J. Struct. Biol.* **116**, 71–76 (1996).
6. Mao, Y. & Finnemann, S. C. Analysis of photoreceptor outer segment

phagocytosis by RPE cells in culture. *Methods Mol. Biol.* **935**, 285–95 (2013).

7. Naldini, L. *et al.* In Vivo Gene Delivery and Stable Transduction of Nondividing Cells by a Lentiviral Vector Published by : American Association for the Advancement of Science Stable URL : http://www.jstor.org/stable/2889637 Accessed : 06-04-2016 15 : 36 UTC Your use of the. *Science (80-.).* **272**, 263–267 (1996).
8. Matet, A. *et al.* Evaluation of tolerance to lentiviral LV-RPE65 gene therapy vector after subretinal delivery in non-human primates. *Transl. Res.* **188**, 40-57.e4 (2017).
9. Bemelmans, A. P. *et al.* Lentiviral gene transfer of Rpe65 rescues survival and function of cones in a mouse model of leber congenital amaurosis. *PLoS Med.* **3**, 1892–1903 (2006).
10. Kostic, C. *et al.* Activity analysis of housekeeping promoters using self-inactivating lentiviral vector delivery into the mouse retina. *Gene Ther.* **10**, 818–821 (2003).
11. Delzor, A. *et al.* Restricted transgene expression in the brain with cell-type specific neuronal promoters. *Hum. Gene Ther. Methods* **254**, 121017063203000 (2012).
